# Iterative sure independence screening EM-Bayesian LASSO algorithm for multi-locus genome-wide association studies

**DOI:** 10.1371/journal.pcbi.1005357

**Published:** 2017-01-31

**Authors:** Cox Lwaka Tamba, Yuan-Li Ni, Yuan-Ming Zhang

**Affiliations:** 1 State Key Laboratory of Crop Genetics and Germplasm Enhancement, Nanjing Agricultural University, Nanjing, China; 2 Department of Mathematics, Egerton University, Egerton, Kenya; 3 Statistical Genomics Lab, College of Plant Science and Technology, Huazhong Agricultural University, Wuhan, China; University of California Irvine, UNITED STATES

## Abstract

Genome-wide association study (GWAS) entails examining a large number of single nucleotide polymorphisms (SNPs) in a limited sample with hundreds of individuals, implying a variable selection problem in the high dimensional dataset. Although many single-locus GWAS approaches under polygenic background and population structure controls have been widely used, some significant loci fail to be detected. In this study, we used an iterative modified-sure independence screening (ISIS) approach in reducing the number of SNPs to a moderate size. Expectation-Maximization (EM)-Bayesian least absolute shrinkage and selection operator (BLASSO) was used to estimate all the selected SNP effects for true quantitative trait nucleotide (QTN) detection. This method is referred to as ISIS EM-BLASSO algorithm. Monte Carlo simulation studies validated the new method, which has the highest empirical power in QTN detection and the highest accuracy in QTN effect estimation, and it is the fastest, as compared with efficient mixed-model association (EMMA), smoothly clipped absolute deviation (SCAD), fixed and random model circulating probability unification (FarmCPU), and multi-locus random-SNP-effect mixed linear model (mrMLM). To further demonstrate the new method, six flowering time traits in *Arabidopsis thaliana* were re-analyzed by four methods (New method, EMMA, FarmCPU, and mrMLM). As a result, the new method identified most previously reported genes. Therefore, the new method is a good alternative for multi-locus GWAS.

## Author summary

Genome-wide association study is concerned with the associations between markers and traits of interest so as to identify all the significantly associated markers. In genome-wide association studies, hundreds of thousands of markers are genotyped for several hundreds of individuals. Usually, only a very minor subset of these markers is associated with the trait. Most penalization methods fail when the number of markers is much larger than the sample size. Based on this fact, we have developed an algorithm that proceeds in two stages. In the first stage (screening), we reduced the number of markers via correlation learning to a moderate size. We then used a moderate-scale variable selection method to select variables in the reduced model. Conditional on the selected variables, we repeated the screening procedure and chose another set of variables. In the second stage (estimation), all the above-selected variables are accurately estimated in a multi-locus model. Our approach is simple, accurate in estimation, fast and shows high statistical power of detecting relevant markers on simulated data. We have also used this method to identify relevant genes in real data analysis. We recommend our approach for conducting a multi-locus genome-wide association study.

## Introduction

Genome-wide association study (GWAS) focuses on associations between single nucleotide polymorphism (SNP) and traits of interest in order to investigate the genetic foundation of these traits [1, 2]. In GWAS, hundreds of thousands of SNPs are genotyped for several hundreds of individuals. In this case, statistical estimation and detection of the relationship between these SNPs and the traits become challenging. Although the single variant analysis in standard GWAS methods has succeeded in identifying thousands of genetic variants associated with hundreds of various traits, this approach fails to consider the joint effect of multiple genetic markers on traits. Another problem with this approach is the issue of multiple test correction for the threshold value of significance test. The Bonferroni correction is too stringent, and many relevant loci are missed out.

In genetics, only a small subset of SNPs is associated with the phenotype of a trait. This is an example of a variable selection problem for high-dimensional data, where the number of SNPs (*p*) is several times larger than the number of individuals (*n*) [3]. To solve this issue, many penalization methods have been developed in statistics, for example, bridge [4], nonnegative garotte [5], least absolute shrinkage and selection operator (LASSO) [6], smoothly clipped absolute deviation (SCAD) [7], elastic net [8], fused LASSO [9], adaptive LASSO [10] and minimax concave penalty [11]. Among these methods, Bayesian LASSO [12], penalized logistic regression [13], sure independence screening [14], adaptive mixed LASSO [15], elastic net [16], LASSO [17], empirical Bayes [18] and empirical Bayes LASSO [19] have been adopted in GWAS. These methods are multi-locus in nature, hence a less stringent significance criterion can be adopted [20]. Despite these methods being able to shrink some markers to zero, they will fail when the number of markers is several times larger than the sample size. In this case, the solution lies in reducing the number of markers before employing a shrinkage method in the multi-locus genetic model. For example, a Bayesian sparse linear mixed model [21] and Bayesian mixture models [22]. However, the computing time becomes a major concern for these Bayesian approaches. An alternative is to integrate single-marker scanning with the multi-locus models, such as a model-free approach [23], multi-locus random-SNP-effect mixed linear model (mrMLM) [20], and fixed and random model circulating probability unification (FarmCPU) [24].

In this study, we developed an approach that reduced the number of markers, *p*, via correlation learning (i.e. Iterative modified-Sure Independence Screening) to a moderate number. A moderate-scale variable selection method, SCAD, was then employed to select variables in the reduced model. We chose SCAD because of its nice oracle property. Conditional on the selected variables, we repeated the screening procedure and chose another set of variables. All the effects of the above-selected variables were estimated by Expectation-Maximization (EM)-Bayesian LASSO algorithm [25] and tested by likelihood ratio statistic for true quantitative trait nucleotide (QTN) detection. We call this approach Iterative modified-Sure Independence Screening EM-Bayesian LASSO (ISIS EM-BLASSO) algorithm. A series of simulated and real datasets were used to validate this new method. We compared our new method with single-locus methods: efficient mixed-model association (EMMA) [26] and FarmCPU [24], multi-locus methods: SCAD [7] and mrMLM [20]. Several reasons guided the choice of these comparison methods: EMMA [26] has been a standard gold method for GWAS, FarmCPU [24] reduces the number of markers used in GWAS just like ISIS EM-BLASSO, SCAD [7] is used in the screening method of ISIS EM-BLASSO hence the need to compare it independently and lastly, mrMLM [20] integrates single locus with multi-locus approach.

## Results

### Statistical power for QTN detection

**Fig 1:**
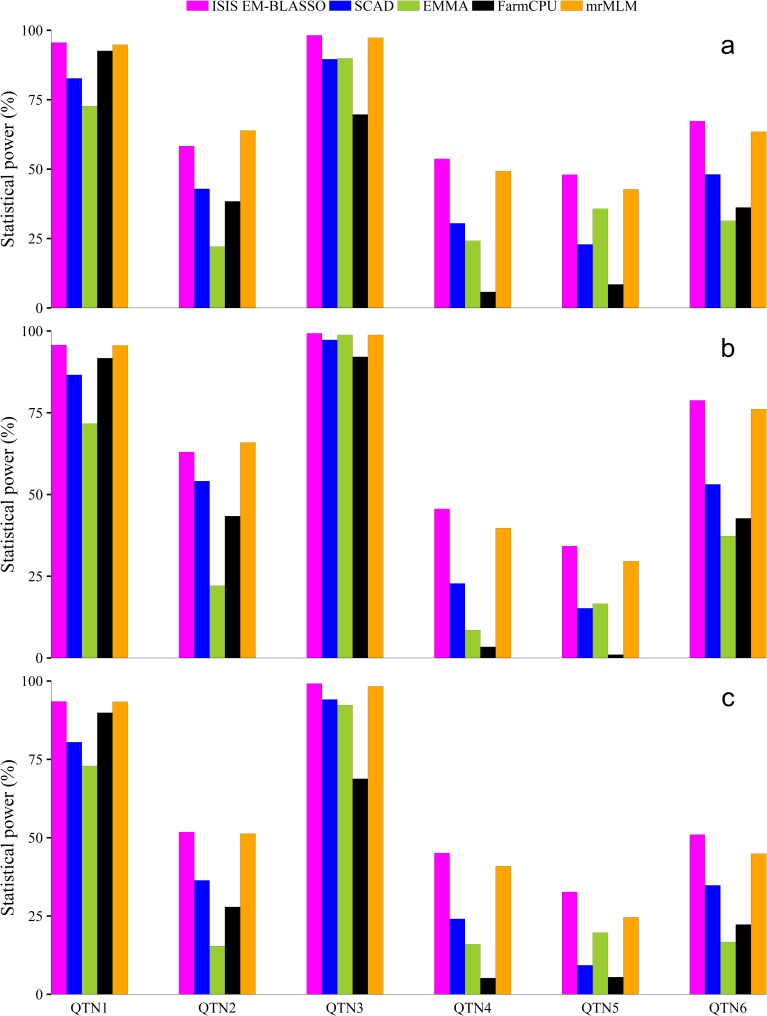
Statistical powers for all the simulated QTNs detected by ISIS EM-BLASSO, SCAD, EMMA, FarmCPU and mrMLM in Monte Carlo simulation experiments 1 (**a**), 2 (**b**) and 3 (**c**).

Three Monte-Carlo simulation experiments were carried out to measure the effectiveness of our new method. We used statistical power to evaluate the effectiveness of ISIS EM-BLASSO method alongside the three methods for comparison purposes. For each QTN, we defined its statistical power as the fraction of the samples in which the QTN was detected (see Methods for significant testing). Fig 1A, 1B and 1C represents the results of the statistical power of detecting each QTN from the three simulation experiments respectively. In the first simulation experiment in which no polygenic variance was simulated, ISIS EM-BLASSO method has the highest power for detecting almost all the six simulated QTNs except QTN two. mrMLM has a high power of detecting the second QTN. EMMA and FarmCPU have a low power of detecting the second and fourth simulated QTNs (Fig 1A and S1 Table). Indeed, Bonferroni correction is too stringent, and it may cause many significant loci to be missed out. SCAD has a moderately higher power than EMMA and FarmCPU for the second, third and sixth QTNs. SCAD lacks consistency in detecting the simulated QTNs hence it cannot be relied upon especially when the QTN size is small. The same trends were observed in the second simulation experiment (Fig 1B and S2 Table) when an additive polygenic variance was added to the polygenic background. In the third simulation experiment (Fig 1C and S3 Table) where three pairs of epistatic effects (collectively contributing 0.15 to the phenotypic variance) were added to the genetic background, ISIS EM-BLASSO is still powerful in detecting almost all the six simulated QTNs. We presented a paired t- test for the differences in statistical power (Table 1). We observe that there are significance differences (at the 0.05 level) in statistical power between ISIS EM-BLASSO and the other three methods (SCAD, EMMA, FarmCPU). There are no significance differences in statistical power between ISIS EM-BLASSO and mrMLM except in the third simulation (at the 0.1 level). Based on these findings, it implies that the simulated QTNs are mostly likely to be identified when ISIS EM-BLASSO method is used.

**Table 1:** The *t* values and their probabilities in paired *t* tests for the differences (A−B) of statistical power or mean squared error (MSE) between ISIS EM-BLASSO (A) and other methods (B) in Monte Carlo simulation experiments.

Case	Simulation experiment	ISIS EM-BLASSO & EMMA		ISIS EM-BLASSO & SCAD		ISIS EM-BLASSO & FarmCPU		ISIS EM-BLASSO & mrMLM	
		t-value	P-value	t-value	P-value	t-value	P-value	t-value	P-value
**Statistical power (%)**	I	6.78	0.0011^***^	4.99	0.0041^***^	4.43	0.0069^***^	0.98	0.3704
	II	3.85	0.0121^**^	4.08	0.0095^***^	3.65	0.0147^**^	1.38	0.2266
	III	5.97	0.0019^***^	4.81	0.0048^***^	5.21	0.0035^***^	2.44	0.0588^*^
**MSE**	I	-7.46	0.0007^***^	-3.77	0.0131^**^	-1.89	0.1167	-0.81	0.4540
	II	-3.98	0.0105^**^	-3.95	0.0109^**^	-1.55	0.1819	-0.19	0.8532
	III	-4.18	0.0086^***^	-3.41	0.0190^**^	-1.48	0.2002	-0.07	0.9447

H_0_: no significance differences (A−B) in statistical power or MSE between ISIS EM-BLASSO (A) and the other method (B). EMMA: efficient mixed model association; SCAD: smoothly clipped absolute deviation; FarmCPU: fixed and random model circulating probability unification; mrMLM: multi-locus random-SNP-effect mixed linear model.

*, ** and ***: statistical significances at the 0.1, 0.05 and 0.01 levels, respectively.

### Accuracies of estimated QTN effects

**Fig 2:**
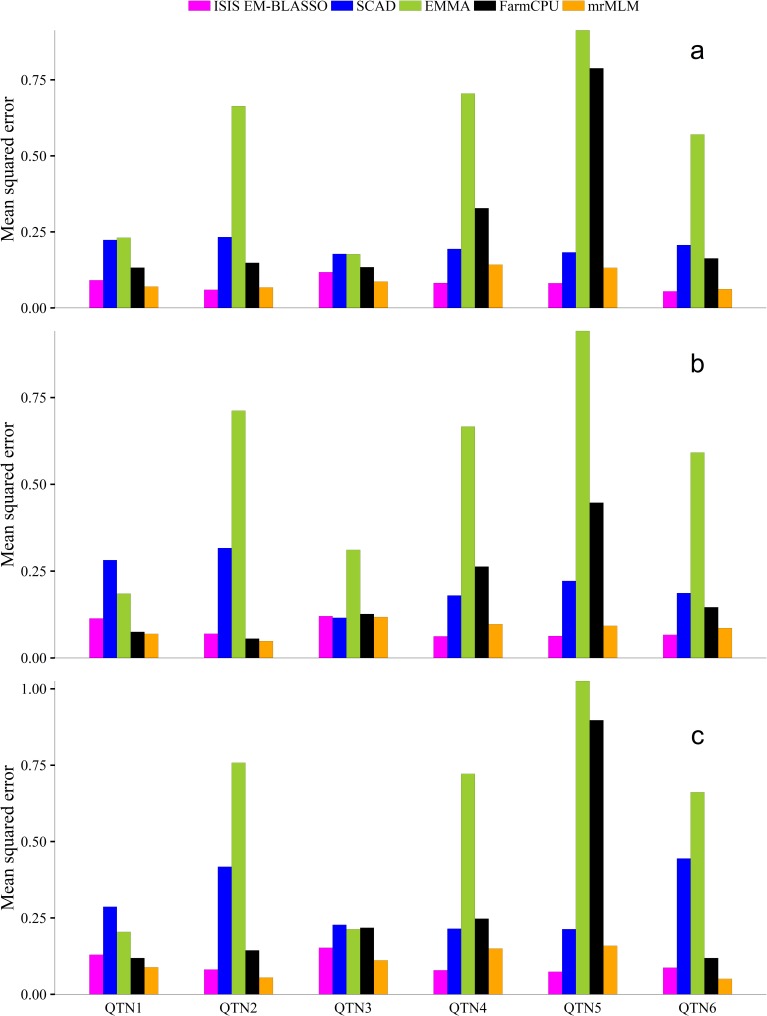
Mean square error (MSE) for all the simulated QTN effects estimated by ISIS EM-BLASSO, SCAD, EMMA, FarmCPU and mrMLM in Monte Carlo simulation experiments 1 (**a**), 2 (**b**) and 3 (**c**).

Mean squared error (MSE) was used to measure the accuracy of each estimated QTN effect for all the methods. We evaluated the accuracies of all the six simulated QTNs effects in the three simulation experiments. The results of all methods considered are presented in Fig 2 and S1, S2 and S3 Tables. The ISIS EM-BLASSO method is consistently more accurate in estimating the QTN effects than the other methods (EMMA, SCAD, and FarmCPU). From these results, EMMA has the highest MSEs for each of six simulated QTNs, implying it is inaccurate in estimating the QTN effect. ISIS EM-BLASSO has lower MSEs than mrMLM for simulated QTNs 4, 5 and 6 in all the three simulations. This implies that it is reliable for estimating the QTN effects. At the 0.05 significance level, the differences of MSEs between ISIS EM-BLASSO and other methods (EMMA and SCAD) were significant (Table 1). Applying EM-Bayesian LASSO to SNPs selected from the iterative procedure will not only remove unimportant SNPs but also improves the effect estimation.

### Empirical type 1 errors and ROC curve

**Fig 3:**
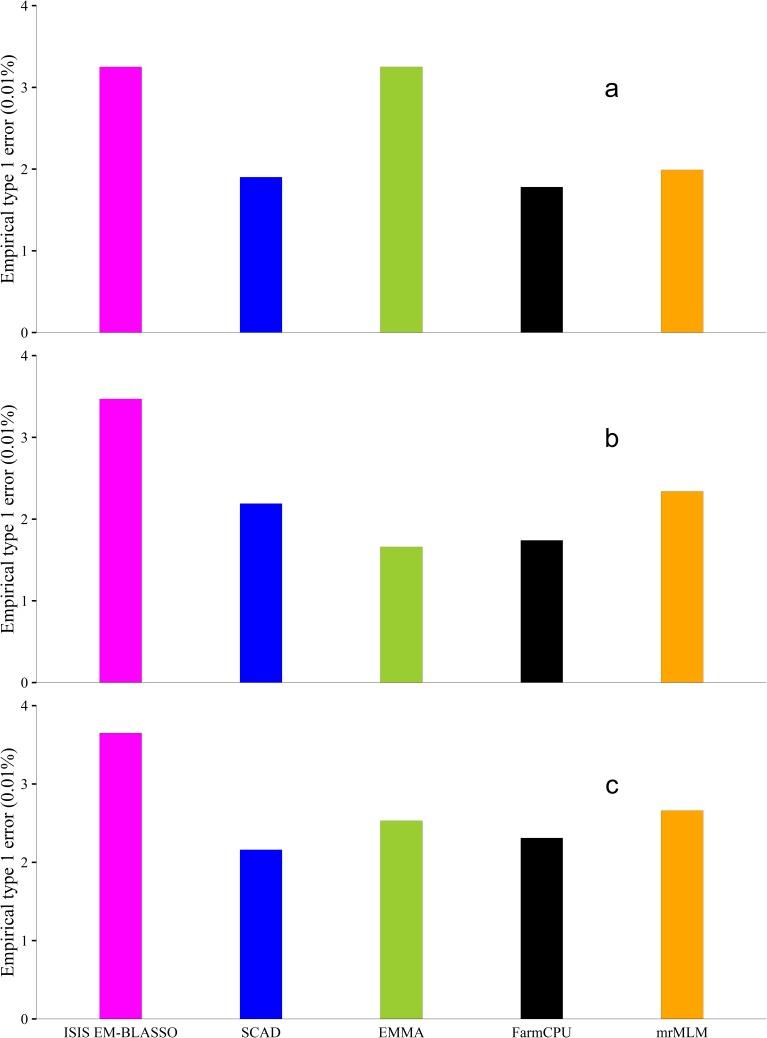
Empirical type 1 error rates derived from ISIS EM-BLASSO, SCAD, EMMA, FarmCPU and mrMLM in the Monte Carlo simulation experiments 1 (**a**), 2 (**b**) and 3 (**c**).

Type 1 errors for all the methods in the three simulation experiments were calculated (Fig 3). The values for Type 1 errors for each method in each simulation are recorded in S1, S2 and S3 Tables. Despite having high power in the detection of QTNs, ISIS EM-BLASSO had slightly higher Type 1 errors compared with SCAD, EMMA, FarmCPU, and mrMLM. Note that all the Type 1 errors were less than 0.04%. Bonferroni correction eliminates many un-associated loci hence reducing the false positive rate in FarmCPU and EMMA at the expense of some associated SNPs. Conversely, this study reveals that ISIS EM-BLASSO method may slightly include some un-important SNPs in the model than SCAD, EMMA, FarmCPU and mrMLM though with a higher power in detecting associated QTNs.

**Fig 4:**
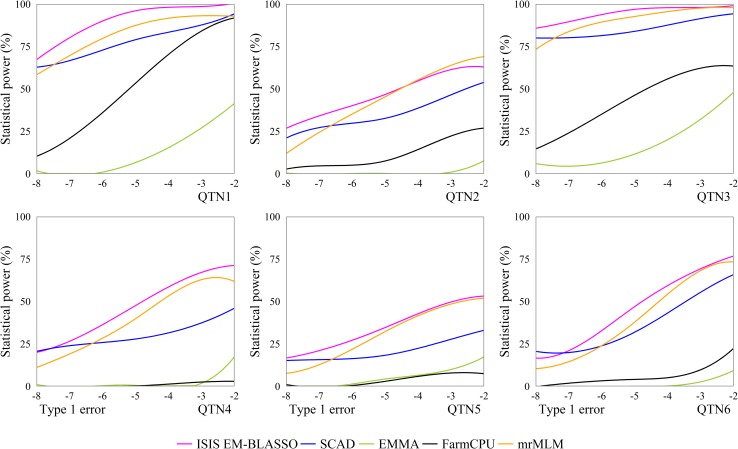
Statistical powers of all the simulated QTNs in the first simulation experiment plotted against Type 1 error (in a log10 scale) for the five GWAS methods (ISIS EM-BLASSO, SCAD, EMMA, FarmCPU, and mrMLM).

Receiver operating characteristic (ROC) curve is used to compare different methods for their efficiencies in the detection of significant effects. ROC curve is a plot of the statistical power against the controlled Type 1 error. A method with the highest ROC curve is deemed the best. We simulated various 67 probability levels of significance between 1e-8 to 1e-2, with these values we calculated the corresponding powers in the first simulation experiment. Fig 4 shows a comparison of the ROC curves from the four methods for the first experiment for each of the six QTNs simulated. ISIS EM-BLASSO performs best among all the other methods considered for all the simulated QTNs.

### Computational efficiency

**Fig 5:**
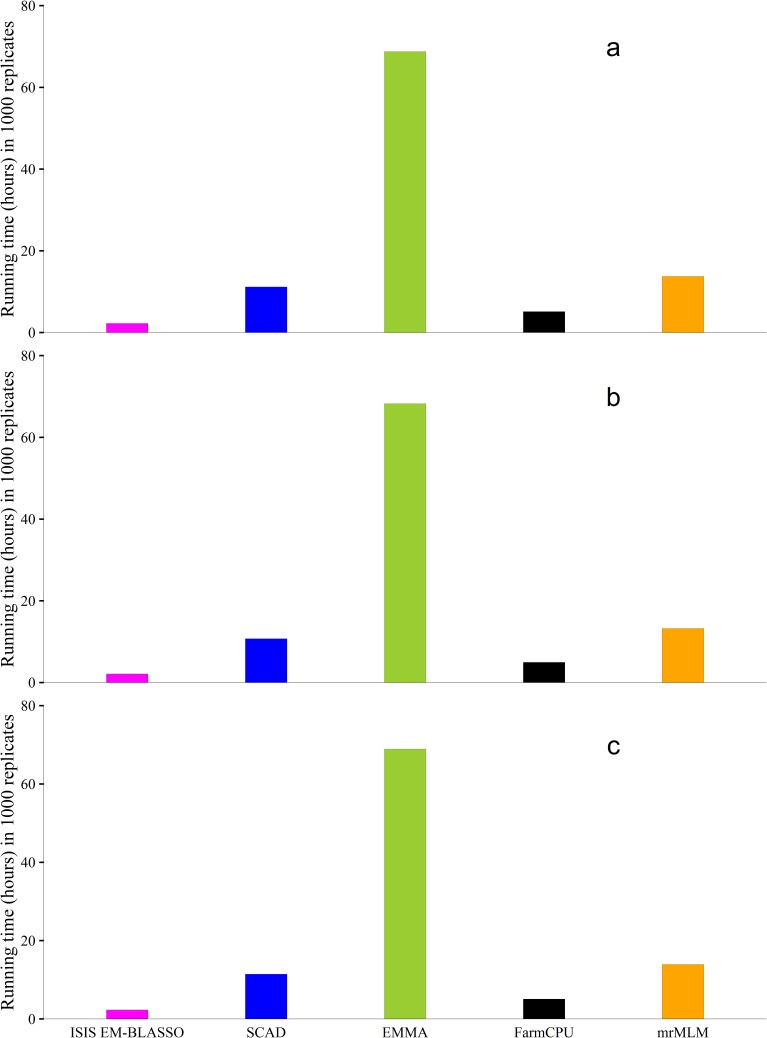
Computing times (hours) in the Monte Carlo simulation experiments 1 (**a**), 2 (**b**) and 3 (**c**).

Comparing the computing times (Fig 5 and S1, S2 and S3 Tables) of these methods in the three simulation experiments respectively, we observed that ISIS EM-BLASSO has the lowest computing time whereas EMMA takes a longer computing time (Intel Core i5-4570 CPU 3.20GHz, Memory 7.88G). ISIS EM-BLASSO is computationally efficient and can be used in GWAS in a few hours to obtain the associated genes. ISIS EM-BLASSO in itself reduces the number of SNPs to only those that are significantly correlated with the phenotype and hence this reduces the problem to a moderate high dimensional data setting problem saving on the computational time.

### Real data analysis in *Arabidopsis*

Six *Arabidopsis* flowering time traits in Atwell et al. [27] have been re-analyzed by ISIS EM-BLASSO, EMMA, FarmCPU, and mrMLM. These traits are LD, LDV, SD, 0W, 2W, and 4W. ISIS EM-BLASSO detected 14, 11, 23, 21, 9 and 11 SNPs to be significantly associated respectively with the six traits above. These detected SNPs for each trait were used to conduct a multiple linear regression analysis, and the corresponding AIC and BIC were calculated. The ISIS EM-BLASSO method showed low AIC and BIC values for nearly all traits (S4 Table). The only method that compares almost equally with ISIS EM-BLASSO is mrMLM. This indicates that SNPs detected by ISIS EM-BLASSO fit the data better than the other methods.

The numbers of known genes in the proximity of SNPs for the above six traits were in total 67 genes from ISIS EM-BLASSO, 22 genes from mrMLM, 15 genes from FarmCPU, and 13 genes from EMMA. ISIS EM-BLASSO detected more known genes than the other methods (S5 Table). Among these known genes, 50 were identified only by ISIS EM-BLASSO (Table 2).

**Table 2:** Known genes identified only by ISIS EM-BLASSO in the GWAS of six flowering time traits in *Arabidopsis thaliana*.

Trait	Gene name	Gene ID	Chr	SNP position (bp)	ISIS EM-BLASSO			Distance to gene (bp)
					r^2^	Effect	LOD	
LD	NTL	AT1G11570	1	3875883	1.65	-0.11	3.95	-7397
	SPL4	AT1G53160	1	19802665	2.47	-0.19	5.68	3754
	VAR2	AT2G30940	2	13143390	2.24	-0.11	6.93	-25143
	EHD2	AT4G05520	4	2819449	0.94	-0.07	3.69	-11520
	APX3	AT4G35000	4	16689198	1.67	-0.10	4.81	-14427
LDV	MOS2	AT1G33520	1	12179065	3.22	-0.05	10.33	20112
	FKF1	AT1G68050	1	25508081	1.51	0.03	5.05	595
	SMG7	AT5G19400	5	6546259	3.06	-0.06	6.66	1244
	ATPI4K*3	AT5G24240	5	8248050	2.45	0.06	5.89	13900
	AGL17	AT2G22630	2	9588685	3.66	-0.05	4.17	-29687
SD	RAV1	AT1G13260	1	4553530	2.32	-0.07	13.61	9788
	EXT3	AT1G21310	1	7448446	0.37	-0.02	3.64	-4790
	AT2G12400	AT2G12400	2	4999298	0.96	-0.04	8.48	-5645
	PAP13	AT2G32770	2	13912889	3.45	-0.10	21.88	14612
	SPA1	AT2G46340	2	19044616	0.63	-0.04	5.65	17203
	PDF1	AT3G25800	3	9437458	0.24	0.03	4.16	11414
	CDG1	AT3G26940	3	9926056	0.65	-0.03	6.38	-10526
	CKL11	AT4G14340	4	8228311	0.24	-0.03	3.90	-19975
	EBS	AT4G22140	4	11707246	1.05	0.06	9.07	-20480
	CESA1	AT4G32410	4	15645842	1.95	-0.07	13.21	-823
	CYCLIN 1	AT4G37490	4	17632478	1.75	-0.05	11.87	8193
	STN8	AT5G01920	5	338648	0.44	-0.03	4.33	-20459
	NAC089	AT5G22290	5	7379995	0.54	-0.05	5.43	2351
0W	FKF1	AT1G68050	1	6371569	0.42	-0.05	3.05	595
	SPDS1	AT1G23730	1	8392979	0.57	0.06	4.39	27286
	SPDS1	AT1G23820	1	8417196	1.23	0.07	7.74	-3069
	ERD15	AT2G41430	2	17285257	0.77	-0.05	4.56	14715
	ADL4	AT3G60190	3	22255698	0.44	-0.05	3.18	7865
	GAI	AT4G02780	4	1228186	2.17	-0.09	8.25	-9581
	NRGA1	AT4G05590	4	2903746	1.25	-0.07	8.73	-3319
	NIP5;1	AT4G10380	4	6415030	0.30	0.04	3.36	-16206
	RAP74	AT4G12610	4	7459086	1.30	0.09	6.32	768
	AGL19	AT4G22950	4	12048235	1.33	-0.07	7.45	20814
	CESA1	AT4G32410	4	15645842	1.97	-0.11	7.70	-823
	HAM4	AT4G36710	4	17325234	0.76	0.0484	5.43	17591
	ATXR5	AT5G09790	5	3037582	0.45	0.04	4.48	-1396
	ROXY2	AT5G14070	5	4867634	1.01	-0.07	5.01	-8881
	SPL7	AT5G18830	5	6289819	0.53	-0.04	4.46	9138
	DGR2	AT5G25460	5	8844702	2.46	-0.09	8.34	-18661
2W	DD46	AT1G22015	1	7758148	2.44	-0.13	5.66	4629
	LPAT3	AT1G51260	1	19008023	1.76	-0.10	3.8131	2382
	ABCB19	AT3G28860	3	10855475	1.77	0.10	3.12	-14563
	ATRBL11	AT3G58460	3	21613189	1.10	-0.09	3.06	-5206
	NGA2	AT3G61970	3	22949227	1.89	0.09	4.43	-2236
4W	ELP	AT1G05850	1	1771680	2.00	-0.11	6.94	2985
	WRKY25	AT2G30250	2	12916879	1.91	-0.09	6.44	11681
	GSTF9	AT2G30860	2	13143876	2.67	-0.12	7.52	3631
	CCT1	AT2G32260	2	13701496	0.71	-0.05	3.42	855
	SMG7	AT5G19400	5	6525973	5.21	-0.19	11.00	-14030
	CKB1	AT5G47080	5	19139239	0.97	0.06	3.64	12586

LD: days to flowering under long days; LDV: days to flowering under long days with vernalization, SD: days to flowering under short days; 0W: days to flowering under LD with no vernalization; 2W and 4W: days to flowering under long days with 2 and 4 weeks vernalized, respectively.

It is interesting to note that for the trait SD, EMMA was not able to determine any significant gene whereas the new approach identified 21 genes. A similar trend is also observed when we considered trait 0W where FarmCPU did not detect any gene. Based on these results, we observe that the new approach can capture the genes associated with the trait under study. We also noted that some genes tested were found significant in nearly all the traits considered. For example, gene *DOGI* was discovered to be associated with LDV, SD, 0W, and 4W, gene *SVP* was found to be related to LD and LDV, gene *ETC3* was found to be associated with LD, 0W, and 2W, and gene *ABCB19* was discovered to be related to SD, 2W, and 4W. These results are consistent with previous studies related to these traits as outlined in the references presented in S5 Table.

Based on the results obtained from this study, we observe that correlation learning can be used as a screening tool to reduce the number of markers in GWAS study. As already noted, most methods used in variable selection in linear regression fail in high and ultra-high dimensional settings. This study has presented a simple yet powerful tool to solve this problem. Therefore, the *Arabidopsis thaliana* GWAS results of this study are reliable.

## Discussion

The single locus tests in standard GWAS methods have been used successfully in identifying thousands of genetic variants associated with hundreds of various traits. As noted by Segura *et al*. [28], when we carry out single-locus tests of association, we risk using the wrong model unless the trait is actually due to a single locus. Single locus tests fail to consider the joint effect of multiple genetic markers on traits. They also suffer from the issue of multiple test correction for the threshold value of significance test. The Bonferroni correction is too stringent, and many significant loci are missed out. This calls for multi-locus testing in GWAS. Because only a subset of SNPs is associated with the phenotype, penalized variable selection methods are appropriate in GWAS. Several penalized methods have been used in solving this problem although they fail when the number of variables, *p*, is several times larger than the sample size (*n*).

In this article, we developed an algorithm, ISIS EM-BLASSO that screens and significantly reduces the number of SNPs to a moderate number. A moderate-scale variable selection method was used to select variables in the reduced set. We chose SCAD since it is asymptotically oracle efficient. Parameter estimation and significance testing were done in the last stage by applying EM-Bayesian LASSO [25] and likelihood ratio test. This algorithm is based on correlation learning in the screening stage. Our approach lays emphasis on the significance of the correlation between SNPs and the trait of interest in the screening stage. It is only the SNPs that are significantly correlated with the trait that are selected in the screening stage. Hence we do not need to subjectively fix the number of SNPs in the screening stage as in Fan and Lv [14] method. ISIS EM-BLASSO differs with the original sure independence screening method by how screening is done. Secondly, it integrates EM- Bayesian LASSO algorithm [25] in the final stage to select and estimate effects. We compared the results of our new approach with results obtained from EMMA [26], FarmCPU [24] and SCAD [7] methods. The reasons are varied: EMMA [26] has been a standard gold method for GWAS, FarmCPU [24] just like ISIS EM-BLASSO also reduces the number of markers used in GWAS, SCAD [7] is actually used in the screening method of ISIS EM-BLASSO hence the need to compare it independently and mrMLM [20] integrates single locus with multi-locus approach. ISIS EM-BLASSO is the fastest as compared to these other methods. ISIS EM-BLASSO only takes 3% of the computing time needed by the EMMA method, 16% of the time taken by mrMLM, 20% of the time taken by SCAD and 50% of the time taken by FarmCPU methods. Screening stage reduces the number of SNPs to an average number hence less time taken in the overall process. More importantly, ISIS EM-BLASSO performs generally the best; it has high statistical power, low Type 1 error and low MSE of estimated QTN effects (S1, S2 and S3 Tables). This algorithm improves the estimation of parameters because even in the last stage, EM-Bayesian LASSO still performs variable selection shrinking other SNPs to zero hence significantly improving the estimates and empirical power. Notice that EM-Bayesian LASSO [25] performs effectively in the last stage because the number of SNPs has been reduced considerably. Just like SCAD, EM-Bayesian LASSO will fail when the number of SNPs runs into hundreds of thousands. For this reason we did not perform tests for EM-Bayesian LASSO independently. Combining sure independence screening, SCAD, and EM-Bayesian LASSO improves the results regarding of empirical power, accuracy, and computational efficiency.

In the screening stage of ISIS EM-BLASSO, iterative sure independence screening can be performed several times. In the article, we only performed a single iteration since we chose a high level of significance, 0.01, for identifying predictors that are significantly correlated with the response. At this level of significance, we expect to have moderately more variables in the screening stage so that SCAD can be applied to shrink some of these variables to zero. A lower level of significance and/or multiple test correction in the screening stage might be too stringent and may result in missing significant SNPs at this juncture. Most shrinkage methods will still perform effectively even when the number of variables, *p*, is moderately greater than or equal to the number of observations, *n*. Therefore the choice of a level of significance of 0.01 without any multiple test correction is justified. Note that if several iterations are performed, then many unimportant variables are selected. Even so, this algorithm is still valid because the extension of this method with EM-Bayesian Lasso [25] in the last stage eliminates un-associated. Other multi-locus GWAS approaches such as multi-locus mixed model (MLMM) [28] have been studied before. MLMM approach of Segura *et al*. [28] is inadequate since its greedy forward-backward method inclusion of SNPs is clearly limited in exploring the huge model space.

ISIS EM-BLASSO considers the joint effect of all SNPs that passes the screening criterion. Unlike EMMA and FarmCPU, we do not apply the Bonferroni correction for multiple testing hence ISIS EM-BLASSO performs better than these methods regarding statistical power. Bonferroni correction is indeed too stringent hence it removes some significant SNPs in the final results when EMMA and FarmCPU are used. Although we still used a slightly stringent criterion of LOD value 3 in our final stage, ISIS EM-BLASSO still has high statistical power and low false positive rate, indicating a better performance of the new algorithm over EMMA and FarmCPU. mrMLM compares almost equally with ISIS EM-BLASSO regarding power and MSE (S1, S2 and S3 Tables). mrMLM is also a two stage method hence the similarity. Nevertheless, ISIS EM-BLASSO is still faster than mrMLM. The results obtained here also demonstrate that many penalized methods fail when the number of SNPs is many times larger than sample size as seen from results obtained when SCAD is used to analyze data.

ISIS EM-BLASSO was used to analyze six flowering time traits in this study. As a result, 67 known genes were detected. Among these known genes, 50 were identified only by ISIS EM-BLASSO (Table 2). Many genes obtained by our approach are in the neighborhood of the 89 SNPs detected (S5 Table). These results are consistent with those previously reported, as shown in the database (http://www.arabidopsis.org/) the work of Atwell et al. [27] and other related references in S5 Table. Atwell et al. [27] listed many significantly associated SNPs to these traits though some of them were not significant at the 0.05/*m* criterion. Therefore, the *Arabidopsis thaliana* GWAS results presented by our algorithm in this study are reliable.

## Conclusion

We considered an iterative modified sure independence screening (ISIS) and extended it by applying EM Bayesian LASSO algorithm herein referred to as ISIS EM-BLASSO. This approach was used to identify relevant genes in GWAS study in real data. The new approach is simple, fast and shows high statistical power of detecting relevant SNPs on simulated data. Mean squared errors of the estimated effects are also minimal. We recommend this approach as an accurate and fast alternative for carrying out multi-locus GWAS study especially in high dimensional settings. ISIS EM-BLASSO reduces the search to a moderate number of SNPs that are significantly correlated with the trait of interest. As a result, we reduce the computing time for GWAS, and also ensure that GWAS can be carried out on a small computer. Our extension by EM-Bayesian LASSO ensures that the parameter estimates are reliable.

## Methods

### Genetic model

In this study, we considered the regression model,

$$y=\mu +\sum_{j=1}^{q}Q_{j}\alpha_{j}+\sum_{i=1}^{p}X_{i}\beta_{i}+\varepsilon$$ (1)

with **y** being a *n*×1 vector of phenotypic quantitative trait, *μ* is the overall average, *Q*_*j*_ is the *jth* fixed effect which must be included in the model, for example, the population structure, **X**_*i*_ is a *n*×1 vector of the *ith* SNP values and *ε*∼MVN_*n*_(0,*σ*^2^**I**) is the residual error.

### Screening and estimation

Given the model in Eq (1) we corrected **y** for the fixed effects *Q*_*j*_, *j* = 1,2,…,*q* before applying the screening procedure. The effects ${\hat{\alpha }}_{j}$ are obtained by ordinary least squares. Eq (1) then becomes, $y_{c}=y-\sum_{j=1}^{q}Q_{j}\alpha_{j}=\mu +\sum_{i=1}^{p}X_{i}\beta_{i}+\varepsilon$ and without loss of generality, this can just be denoted as

$$y=\mu +\sum_{i=1}^{p}X_{i}\beta_{i}+\varepsilon$$ (2)

#### SCAD

All regularized regression methods aim at estimating the vector **β** of regression coefficients in Eq (2) by minimizing an objective function *ξ* composed of a loss function (e.g. residual sum of squares (RSS)) and a penalty function

$$\xi_{\lambda ,\gamma }\left(\beta \right)=\underset{\beta }{\arg\;\min}\{\underset{loss\;function}{\underset{︸}{\sum_{i=1}^{n}{\left(y_{i}-\sum_{j=1}^{p}\left(\beta_{j}X_{ij}\right)\right)}^{2}}}+\underset{penalty\;function}{\underset{︸}{\sum_{j=1}^{p}\rho_{\lambda ,\gamma }\left(\left|\beta_{j}\right|\right)}}\}$$ (3)

where *ρ*_*λ*,*γ*_(⋅) is a function of the vector of coefficients **β** = (*β*_1_,*β*_2_,⋯,*β*_*p*_)^*T*^ and the tuning (penalty) parameter *λ* > 0 controls the trade-off between minimizing the loss and the penalty term. γ>0 is a shrinkage parameter that determines the order of the penalty function. Notice that we avoided *μ* in Eq (3) because we assume that the input data is standardized making overall mean zero. Minimizing Eq (3) yields a spectrum of solutions depending on the value of λ. The gradient (first derivative) of a penalty function determines how it affects the answer in (3) [29]. Typically, the penalty function *ρ* is symmetric about the origin (*ρ*(0) = 0) and its non-decreasing in the interval (0,∞) [7].

Of great importance to us in this study is the SCAD penalty which is defined as

$$\rho_{\lambda ,\gamma }\left(\beta \right)=\lambda \left|\beta \right|1_{\left\{\left|\beta \right|<\lambda \right\}}+\frac{\gamma \lambda \left|\beta \right|-0.5\left(\beta^{2}+\lambda^{2}\right)}{\gamma -1}1_{\left\{\lambda <\left|\beta \right|\leq \gamma \lambda \right\}}+\frac{\lambda^{2}\left(\gamma^{2}-1\right)}{2\left(\gamma -1\right)}1_{\left\{\left|\beta \right|>\gamma \lambda \right\}}$$ (4)

with *λ* ≥ 0, *λ* > 2 and **1**_{*x*∈*A*}_ being an indicator function. This penalty gradually reduces the penalization rate to zero as |**β**| gets larger. The regularization parameter γ controls the degree of concavity, with smaller γ corresponding to more concave penalty when γ → ∞, SCAD converges to the *L*_1_ penalty. [7] Suggested using *γ* = 3.7 for SCAD. SCAD is asymptotically oracle-efficient [7].

#### EM-Bayesian LASSO (EM-BLASSO)

Xu [25] developed the EM-algorithm for the Bayesian LASSO by considering the linear mixed model

$$Y=\sum_{j=1}^{q}X_{j}\beta_{j}+\sum_{k=1}^{p}Z_{k}\gamma_{k}+\varepsilon$$ (5)

where *β*_*j*_ is the *jth* non-QTN effect, **X**_*j*_ is the corresponding design matrix, *γ*_*k*_ is a vector of SNP effects for locus *k* and **Z**_*k*_ is the corresponding incidence matrix determined by the genotypes of the locus *k*. The dimensions of *γ*_*k*_ and **Z**_*k*_ depend on the number, *m*_*k*_ of genotypes for the locus *k*. The residual error vector *ε* is assumed to be distributed as MVN_*n*_(0,*σ*^2^**I**_*n*_). In our case **X** will include the overall mean and the population structure matrix we will use the original before **Y** correction. Using a normal prior for *γ*_*k*_, i.e., $\gamma_{k}∼{\text{MVN}}_{m_{k}}\left(0,\sigma_{k}^{2}I_{m_{k}}\right)$ and two different types of priors of $\sigma_{k}^{2}$, one can solve for ${\hat{\sigma }}_{k}^{2}$ [25]. As for the fixed effects, we have [30],

$$\hat{\beta }={\left(X^{T}V^{-1}X\right)}^{-1}X^{T}V^{-1}Y$$

where $V=\sum_{k=1}^{p}Z_{k}Z_{k}^{T}\sigma_{k}^{2}+I\sigma^{2}$ is the variance component and residual variance estimator is

$${\hat{\sigma }}^{2}=\frac{1}{n}{\left(Y-X\beta \right)}^{T}\left\{\left(Y-X\beta \right)-\sum_{k=1}^{p}Z_{k}E\left(\gamma_{k}\right)\right\}$$ (6)

where *E*(*γ*_*k*_) = *E*(*γ*_*k*_|*θ*,*y*). The EM steps of Xu [25] are developed by letting *γ*_*k*_ be the missing values and by using the following expectations for maximization step.

$$\begin{array}{l} E\left(\gamma_{k}\right)=\sigma_{k}^{2}Z_{k}^{T}V^{-1}\left(Y-X\beta \right)\\ E\left(\gamma_{k}^{T}\gamma_{k}\right)=E\left(\gamma_{k}^{T}\right)E\left(\gamma_{k}\right)+tr\left[\mathrm{var}\left(\gamma_{k}\right)\right]\\ \mathrm{var}\left(\gamma_{k}\right)=I\sigma_{k}^{2}-\sigma_{k}^{2}Z_{k}^{T}V^{-1}Z_{k}\sigma_{k}^{2} \end{array}$$ (7)

#### Iterative Sure Independence Screening-EM Bayesian LASSO (ISIS EM-BLASSO)

We describe a multi-stage approach for screening and selecting relevant variables/SNPs. Our approach first reduces the number of variables via correlation learning approach to a moderate number that can be handled by any moderate-scale variable selection method. In the screening stage, we first reduce the number of SNPs by selecting only SNPs/predictors that are significantly correlated with the response. Applying SCAD method, we select relevant variables while shrinking others to zero. Notice that this is a modified version of sure independence screening (SIS) in Fan and Lv [14]. Herein, our emphasis is on the significance of correlation. In the next stage, we find significant predictors that are marginally uncorrelated with a response. Using an iterative modified sure independence screening (ISIS) procedure, we repeat the SIS procedure conditional on the previously selected variables so as to capture essential variables that are marginally uncorrelated with a response. Notice that a combination of variables selected from the above two steps may not be regarded as a set of significant variables since the false detection rate will be extremely high. Therefore, we have extended this approach by applying EM-Bayesian LASSO algorithm [25] in the final stage. Parametric estimation and significance tests of variables are performed at this last stage. We call this algorithm ISIS EM-BLASSO. We describe the ISIS EM-BLASSO briefly in two stages as follows;

1. **Screening** **Stage**
  - We correct the phenotypes using fixed effects that must be included in the model such as the population structure. We center each input variables so that the observed mean is 0, and scale each predictor so that the sample standard deviation is 1. Let *K*^*T*^ = {1 ≤ *i* ≤ *p*: *β*_*i*_ ≠ 0} be the exact sparse model of size *k* = |*K*^*T*^|. The other *p*−*k* variables can also be correlated with the response variable via linkage to the predictors that are contained in the model.
  - We let **r** = {*r*_1_,*r*_2_,⋯,*r*_*p*_} be a *p*-vector that is obtained by component-wise regression, i.e.,
    $$r=X^{\prime }Y$$ (8)
    with data matrix **X** first being standardized column-wise. **r** is a vector of marginal correlations of predictors with the response variable, rescaled by the standard deviation of the response.
  - By sorting only significant marginal correlation, we define a sub-model,
    $$K_{1}=\{1\leq i\leq p:\;\left|r_{i}\right|\;\text{is significant at the}\;0.01\;\text{level}\}$$ (9)
    We chose to use a significance level of 0.01. At a significant level of 0.01, we will capture even slight correlations between predictors and the response. By sparsity, we expect that the actual model *K*^*T*^ has a size less than *n* hence any moderate shrinkage method will remove unimportant predictors. SCAD is a moderate-scale variable selection method since it selects variables from moderately high dimensional linear model shrinking the unimportant ones to zero. Hence, we applied penalty in Eq (4) to the sub-model *K*_1_ and selected the variables related to the response with a high probability i.e. *K*_1_ ⊇ *K*^*T*^. ncvreg package in R language downloaded from http://cran.r-project.org/web/packages/ncvreg/ facilitates this step. This step is a modified version of SIS-SCAD in Fan and Lv [14].
  - To screen variables that may not be marginally correlated with the response, an iterative modified sure independence screening (ISIS) method is applied. After the modified SIS-SCAD (step (iii) above) has been implemented, we obtain the selected variables of say size *τ*_1_. Then we have an *n*-vector of residuals from regressing the response **Y** over $T_{1}=\left\{X_{i1},X_{i2},\cdots ,X_{i\tau_{1}}\right\}$. Treating the residuals as the new responses, we apply the same procedure in steps (i)–(iii) to the remaining *p*−*τ*_1_ variables. This results in another subset of size, say *τ*_2_ of variables in the sub-model $T_{2}=\left\{X_{j1},X_{j2},\cdots ,X_{j\tau_{2}}\right\}$. This results in a selected sub-model of size, say *τ* = *τ*_1_ + *τ*_2_. There is a high probability that *K*^*T*^ ⊆ *T*_1_∪*T*_2_.
2. **Estimation** **stage**
  We apply EM-Bayesian LASSO algorithm [25] to filter out and estimate the true effects from the set *T*_1_∪*T*_2_ of size *τ* = *τ*_1_ + *τ*_2_. Using the model,
  $$Y=X\beta +\sum_{k=1}^{\tau }Z_{k}\gamma_{k}+\varepsilon$$ (10)
  we estimate *γ*_*k*_ *for k* = 1,2,…,*τ* with the EM steps being updated using Eqs (6) and (7) till convergence occurs so that ${\hat{\gamma }}_{k}=E\left(\gamma_{k}\right)$ and *Var*(*γ*_*k*_) is the prediction error for *γ*_*k*_. Notice that at this stage, we use the original **Y** before correction, so that *Z*_*k*_ denotes the *kth* SNP values and **X** includes overall mean and population structure.

**Fig 6:**
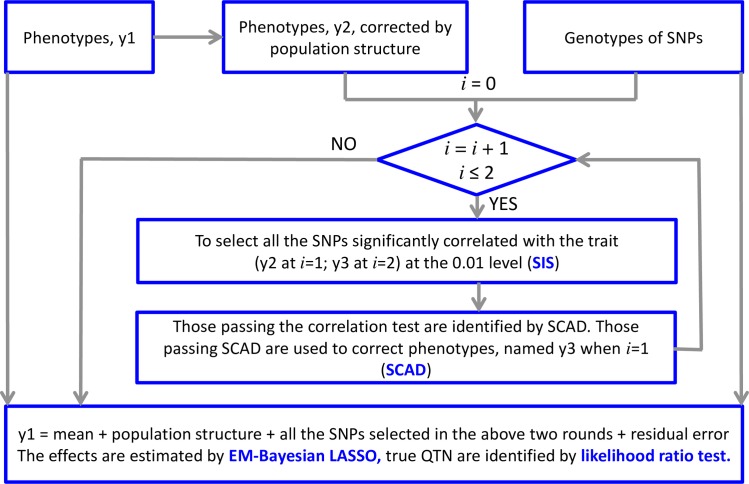
Diagrammatic representation of ISIS EM-BLASSO algorithm.

A diagrammatic representation of our overall algorithm is presented in Fig 6. The new algorithm has been implemented in R and its software can be downloaded from https://cran.r-project.org/web/packages/mrMLM/index.html. For comparison purposes, we have compared ISIS EM-BLASSO with four methods, which are EMMA (http://mouse.cs.ucla.edu/emma/) [26], SCAD (http://cran.r-project.org/web/packages/ncvreg/) [7], mrMLM (http://cran.r-project.org/web/packages/mrMLM/index.html) [20], and FarmCPU (http://www.ZZLab.net/FarmCPU) [24].

### Significance testing

ISIS-EM-BLASSO gives the marker effects estimates, *γ*_*k*_ which must be tested for their significance to the phenotype under study. We propose that all ${\hat{\gamma }}_{k}\neq 0$ *k* = 1,2,⋯,*p* could be viewed to be associated with the trait under study. With the model (10) above at hand, and all ${\hat{\gamma }}_{k}\neq 0$ let’s say of size *q*, we consider the model’s likelihood functions. Let $L_{0}=L\left({\hat{\theta }}_{-k}\right)$ and $L_{1}=L\left({\hat{\theta }}_{1}\right)$ be short expressions of the natural logarithms of the likelihood functions under the null model and the full model, respectively, with ${\hat{\theta }}_{-k}=\left\{\gamma_{\left(1\right)},\cdots ,\gamma_{\left(k-1\right)},\gamma_{\left(k+1\right)},\gamma_{\left(q\right)}\right\}$ and ${\hat{\theta }}_{1}=\left\{\gamma_{\left(1\right)},\cdots ,\gamma_{\left(q\right)}\right\}$.

In essence, we test the null hypothesis, *H*_0_:*γ*_(*k*)_ = 0 that there is no QTN linked to the marker *k*. We use log of odds (LOD) score as the test statistic. The original likelihood functions (before taking the natural log) are $l_{0}=e^{L_{0}}$ and $l_{1}=e^{L_{1}}$, respectively. The LOD score is defined as

$$LOD=\log_{10}(\frac{l_{0}}{l_{1}})=\frac{-2(L_{0}-L_{1})}{4.6052}$$ (11)

The LOD score is easy to interpret because of base 10. We select all markers with a score *LOD* ≥ 3 and regard them as significant. Note that LOD value 3 is a slightly stringent criterion and is equivalent to *p*-value: Pr(*χ*^2^ > 3×4.6052) = 0.0002. If the null hypothesis is true, *LOD* × 4.6052 follows a Chi-square distribution with one degree of freedom. A similar procedure is used to test the significance of the estimates obtained from SCAD and mrMLM.

It is important to point out that, for the EMMA and FarmCPU methods we select significant markers based on Bonferroni correction for multiple tests by setting a threshold for P-value at 0.05/*m*, where *m* is the number of markers.

### Numerical studies and application

#### Monte Carlo simulation experiments

In our simulation studies, all the genetic values for SNP markers were randomly sampled from Atwell et al. [27] (http://www.arabidopsis.org/) and all the phenotypes values for quantitative traits were simulated. The dataset of Atwell et al. [27] includes 199 diverse inbred lines each with 216,130 SNPs. From these SNPs in Atwell et al. [27], we obtained 10000 SNP genotypes by sampling 2000 SNPs randomly from each chromosome. The positions and genotypes for all the selected SNPs were same as those in Wang et al. [20].

In our first simulation study, the dataset consisted of 10000 SNP genotyped from 199 inbred lines. We set six QTN and placed them on the SNPs with allelic frequencies of 0.30; the heritabilities were set as 0.10, 0.05, 0.05, 0.15, 0.05 and 0.05, respectively. The positions and effects of these QTN are listed in S1, S2 and S3 Tables. We set the mean at 10 and variance of random errors at 10. In our simulation study, we applied the ISIS EM-BLASSO, EMMA, SCAD, mrMLM and FarmCPU methods in 1000 runs. For each simulated QTN, we counted the samples in which *LOD* ≥ 3 for ISIS EM-BLASSO, SCAD and mrMLM whereas for EMMA and FarmCPU we selected all markers with *p*-values less than 0.05/*m* (Bonferroni correction for multiple tests), where *m* is the number of markers. Each QTN within 1kb of the simulated QTN was considered a true QTN. A fraction of the number of such samples over the total number of run (1000) represented the empirical power of this QTN. The false positive rate (FPR) was calculated as a fraction of the number of false positive effects over the total number of zero effects considered in the full model. We calculated the mean squared error (MSE) for each QTN to measure the bias of QTN effect estimate. We also performed a paired *t*-test for the differences (A–B) of statistical power or MSE between ISIS EM-BLASSO (A) and other methods (B).

In our second simulation study, we investigated the effect of the polygenic (small effect genes) background on ISIS EM-BLASSO. The polygenic effect was simulated by multivariate normal distribution, ${\text{MVN}}_{n}\left(0,K\sigma_{poly}^{2}\right)$ where $\sigma_{poly}^{2}$ is the polygenic variance, and **K** kinship coefficient matrix between a pair of individuals. Here $\sigma_{poly}^{2}=2$ hence $h_{poly}^{2}=0.092$. The QTN size (*h*^2^), average, residual variance, and other values were the same as those in the first simulation study.

In our third simulation study, we studied the effect of epistatic background on ISIS EM-BLASSO. Three epistatic QTN each with $\sigma_{epi}^{2}=1.25$ and $h_{epi}^{2}=0.05$ were simulated. The details for the three epistatic QTN were described in Wang et al. [20]. The QTN size (*h*^2^), average, residual variance, and other values were the same as those in the first simulation study.

All the above simulated datasets are saved in S1 Dataset.

#### Real data analysis

Six flowering time-related traits in Arabidopsis [27] were analyzed by the ISIS EM-BLASSO, EMMA, and FarmCPU methods to validate the new method. These traits are days to flowering under long days (LD), days to flowering under long days with vernalization (LDV), days to flowering under short days (SD), days to flowering under LD with no vernalization (0W), days to flowering under long days with 2 weeks vernalized (2W) and days to flowering under long days with 4 weeks vernalized (4W). These datasets were downloaded from http://www.arabidopsis.usc.edu/. In the real data analyses, the significantly associated SNPs were determined by the critical threshold of *LOD* ≥ 3 for the new method, SCAD, and mrMLM, and with the P-value less than 0.05/*m* for EMMA and FarmCPU. Candidate genes for the trait under study were mined within 30 kb of the significantly associated SNP.

## Supporting information

S1 Table Comparison of ISIS EM-BLASSO (new), EMMA, SCAD, FarmCPU and mrMLM in the first simulation experiment without polygenic background. (DOC)

S2 Table Comparison of ISIS EM-BLASSO (new), EMMA, SCAD, FarmCPU and mrMLM in the second simulation experiment with an additive polygenic background (explaining 0.092 of the phenotypic variance). (DOC)

S3 Table Comparison of ISIS EM-BLASSO (new), EMMA, SCAD, FarmCPU and mrMLM in the third simulation experiment with three epistatic QTNs each explaining 0.05 of the phenotypic variance. (DOC)

S4 Table Goodness of fit (AIC, BIC) for SNPs detected by four methods (ISIS EM-BLASSO (new), EMMA, FarmCPU, and mrMLM), where a lower value indicates a better fit. (DOC)

S5 Table Genome-wide association studies for six flowering time traits in *Arabidopsis thaliana* using ISIS EM-BLASSO (new), EMMA, FarmCPU, and mrMLM. (DOC)

S1 Dataset All the genetic values for SNP markers were randomly sampled from Atwell et al. [1] (2010) (“Genotypes” sheet) and all the phenotypes values for quantitative traits were simulated (Phenotypes_1, Phenotypes_2 and Phenotypes_3 sheets). In the “Genotypes” sheet, there are 199 diverse inbred lines, each with 10000 SNP genotypes, obtained by sampling 2000 SNPs randomly from each chromosome in Atwell et al. [27] (http://www.arabidopsis.org/). In the “Phenotypes_*” sheet, all the phenotypic values with 1000 replicates were simulated based on genotypic values in “Genotypes” sheet and simulated parameters. The *i*th sample consists of the (199(*i*−1)+1)th to 199*i*th rows in the first column (*i* = 1,…,1000). “*” indicates the sequence number (1, 2 and 3) of simulated experiments.
(XLSX)
