# Supplementary material for: Iterative sure independence screening EM-Bayesian LASSO algorithm for multi-locus genome-wide association studies
Source: PLoS Comput Biol. 2017 Jan 31;13(1):e1005357. doi: 10.1371/journal.pcbi.1005357 (PMC5308866; doi:10.1371/journal.pcbi.1005357)
Supplement: S1 Table — (DOC) [file pcbi.1005357.s001.doc]

### S1 Table: Comparison of ISIS EM-BLASSO (new), EMMA, SCAD, FarmCPU and mrMLM in the first simulation experiment without polygenic background

| **QTN** | **True values** | | | | **ISIS EM-BLASSO** | | | **EMMA** | | | **SCAD** | | | **FarmCPU** | | | **mrMLM** | | |
| --- | --- | --- | --- | --- | --- | --- | --- | --- | --- | --- | --- | --- | --- | --- | --- | --- | --- | --- | --- |
|  | **Position (bp)** | **Chr** | **r^2^** | **Effect** | **Effect** | **MSE** | **Power** | **Effect** | **MSE** | **Power** | **Effect** | **MSE** | **Power** | **Effect** | **MSE** | **Power** | **Effect** | **MSE** | **Power** |
| 1 | 11298364 | 1 | 0.1 | 1.476 | 1.4044 | 0.0913 | 0.956 | 1.9084 | 0.2309 | 0.727 | 1.2574 | 0.2236 | 0.827 | 1.7089 | 0.1327 | 0.926 | 1.5412 | 0.0701 | 0.948 |
| 2 | 11655607 | 1 | 0.05 | 1.044 | 1.0847 | 0.0598 | 0.583 | 1.8464 | 0.6634 | 0.221 | 0.7913 | 0.2330 | 0.429 | 1.3577 | 0.1484 | 0.384 | 1.2083 | 0.0674 | 0.639 |
| 3 | 5134228 | 2 | 0.15 | 1.808 | 1.6819 | 0.1177 | 0.982 | 2.1590 | 0.1766 | 0.899 | 1.6473 | 0.1777 | 0.896 | 2.0414 | 0.1341 | 0.697 | 1.7922 | 0.0867 | 0.973 |
| 4 | 5066968 | 2 | 0.05 | 1.044 | 1.1419 | 0.0821 | 0.537 | 1.8675 | 0.7049 | 0.242 | 1.0802 | 0.1941 | 0.305 | 1.4307 | 0.3279 | 0.058 | 1.3291 | 0.1423 | 0.493 |
| 5 | 5464675 | 2 | 0.05 | 1.044 | 1.1705 | 0.0815 | 0.480 | 1.9862 | 0.9128 | 0.357 | 1.1445 | 0.1829 | 0.229 | 1.8746 | 0.7880 | 0.085 | 1.3174 | 0.1318 | 0.427 |
| 6 | 6137189 | 2 | 0.05 | 1.044 | 1.0484 | 0.0546 | 0.673 | 1.7836 | 0.5704 | 0.314 | 0.7740 | 0.2068 | 0.481 | 1.3858 | 0.1630 | 0.362 | 1.2072 | 0.0619 | 0.635 |
| **Empirical Type 1 Error (0.01%)** | | | | | 3.25 | | | 3.25 | | | 1.90 | | | 1.78 | | | 1.99 | | |
| **Time Taken (Hrs)**  **(199 individuals with 10000 SNPs 1000 replicates)** | | | | | 2.26 | | | 68.77 | | | 11.17 | | | 5.12 | | | 13.77 | | |

Chr: chromosome, r^2^: the proportion of phenotypic variance explained by each QTL, MSE: mean square error.
