# Supplementary material for: Iterative sure independence screening EM-Bayesian LASSO algorithm for multi-locus genome-wide association studies
Source: PLoS Comput Biol. 2017 Jan 31;13(1):e1005357. doi: 10.1371/journal.pcbi.1005357 (PMC5308866; doi:10.1371/journal.pcbi.1005357)
Supplement: S2 Table — (DOC) [file pcbi.1005357.s002.doc]

### S2 Table: Comparison of ISIS EM-BLASSO (new), EMMA, SCAD, FarmCPU and mrMLM in the second simulation experiment with an additive polygenic background (explaining 0.092 of the phenotypic variance)

| **QTN** | **True values** | | | | **ISIS EM-BLASSO** | | | **EMMA** | | | **SCAD** | | | **FarmCPU** | | | **mrMLM** | | |
| --- | --- | --- | --- | --- | --- | --- | --- | --- | --- | --- | --- | --- | --- | --- | --- | --- | --- | --- | --- |
|  | **Position (bp)** | **Chr** | **r^2^** | **Effect** | **Effect** | **MSE** | **Power** | **Effect** | **MSE** | **Power** | **Effect** | **MSE** | **Power** | **Effect** | **MSE** | **Power** | **Effect** | **MSE** | **Power** |
| 1 | 11298364 | 1 | 0.1 | 1.617 | 1.4533 | 0.1137 | 0.957 | 1.9993 | 0.1852 | 0.717 | 1.3013 | 0.2818 | 0.866 | 1.7735 | 0.0751 | 0.917 | 1.5845 | 0.0692 | 0956 |
| 2 | 11655607 | 1 | 0.05 | 1.144 | 1.0804 | 0.0699 | 0.630 | 1.9752 | 0.7121 | 0.221 | 0.7903 | 0.3164 | 0.541 | 1.3270 | 0.0556 | 0.434 | 1.2283 | 0.0484 | 0.659 |
| 3 | 5134228 | 2 | 0.15 | 1.981 | 1.9908 | 0.1206 | 0.993 | 2.4713 | 0.3111 | 0.988 | 2.0463 | 0.1158 | 0.973 | 2.3157 | 0.1267 | 0.921 | 2.0872 | 0.1177 | 0.988 |
| 4 | 5066968 | 2 | 0.05 | 1.144 | 1.1538 | 0.0624 | 0.456 | 1.9486 | 0.6665 | 0.085 | 1.0745) | 0.1799 | 0.228 | 1.5314 | 0.2631 | 0.034 | 1.3408 | 0.0973 | 0.397 |
| 5 | 5464675 | 2 | 0.05 | 1.144 | 1.1371 | 0.0631 | 0.342 | 2.1039 | 0.9415 | 0.166 | 1.0577 | 0.2219 | 0.152 | 1.7980 | 0.4473 | 0.010 | 1.3282 | 0.0924 | 0.296 |
| 6 | 6137189 | 2 | 0.05 | 1.144 | 1.1664 | 0.0665 | 0.788 | 1.8969 | 0.5915 | 0.373 | 0.9534 | 0.1869 | 0.531 | 1.5450 | 0.1460 | 0.427 | 1.3339 | 0.0861 | 0.761 |
| **Empirical Type 1 Error (0.01%)** | | | | | 3.47 | | | 1.66 | | | 2.19 | | | 1.74 | | | 2.34 | | |
| **Time Taken (Hrs)**  **(199 individuals with 10000 SNPs 1000 replicates)** | | | | | 2.12 | | | 68.25 | | | 10.74 | | | 4.95 | | | 13.25 | | |

Chr: chromosome, r^2^: the proportion of phenotypic variance explained by each QTL; MSE: mean square error.
