# Supplementary material for: Iterative sure independence screening EM-Bayesian LASSO algorithm for multi-locus genome-wide association studies
Source: PLoS Comput Biol. 2017 Jan 31;13(1):e1005357. doi: 10.1371/journal.pcbi.1005357 (PMC5308866; doi:10.1371/journal.pcbi.1005357)
Supplement: S3 Table — (DOC) [file pcbi.1005357.s003.doc]

### S3 Table: Comparison of ISIS EM-BLASSO (new), EMMA, SCAD, FarmCPU and mrMLM in the third simulation experiment with three epistatic QTNs each explaining 0.05 of the phenotypic variance

| **QTN** | **True values** | | | | **ISIS EM-BLASSO** | | | **EMMA** | | | **SCAD** | | | **FarmCPU** | | | **mrMLM** | | |
| --- | --- | --- | --- | --- | --- | --- | --- | --- | --- | --- | --- | --- | --- | --- | --- | --- | --- | --- | --- |
|  | **Position (bp)** | **Chr** | **r^2^** | **Effect** | **Effect** | **MSE** | **Power** | **Effect** | **MSE** | **Power** | **Effect** | **MSE** | **Power** | **Effect** | **MSE** | **Power** | **Effect** | **MSE** | **Power** |
| 1 | 11298364 | 1 | 0.1 | 1.731 | 1.5990 | 0.1298 | 0.935 | 2.1286 | 0.2044 | 0.729 | 1.4639 | 0.2867 | 0.805 | 1.9092 | 0.1190 | 0.899 | 1.7479 | 0.0886 | 0.934 |
| 2 | 11655607 | 1 | 0.05 | 1.224 | 1.1238 | 0.0813 | 0.518 | 2.0837 | 0.7576 | 0.154 | 0.7773 | 0.4177 | 0.364 | 1.5270 | 0.1440 | 0.279 | 1.3319 | 0.0550 | 0.513 |
| 3 | 5134228 | 2 | 0.15 | 2.12 | 1.9629 | 0.1526 | 0.992 | 2.5027 | 0.2131 | 0.923 | 1.9636 | 0.2276 | 0.941 | 2.4242 | 0.2179 | 0.688 | 2.1027 | 0.1114 | 0.983 |
| 4 | 5066968 | 2 | 0.05 | 1.224 | 1.2958 | 0.0789 | 0.451 | 2.0656 | 0.7218 | 0.16 | 1.2139 | 0.2149 | 0.241 | 1.3970 | 0.2478 | 0.052 | 1.5137 | 0.1497 | 0.409 |
| 5 | 5464675 | 2 | 0.05 | 1.224 | 1.2847 | 0.0740 | 0.327 | 2.2242 | 1.0254 | 0.197 | 1.2917 | 0.2133 | 0.093 | 2.0753 | 0.8971 | 0.055 | 1.5209 | 0.1590 | 0.246 |
| 6 | 6137189 | 2 | 0.05 | 1.224 | 1.0849 | 0.0872 | 0.510 | 2.0238 | 0.6615 | 0.167 | 0.6802 | 0.4446 | 0.348 | 1.4843 | 0.1189 | 0.223 | 1.2886 | 0.0507 | 0.449 |
| **Empirical Type 1 Error (0.01%)** | | | | | 3.65 | | | 2.53 | | | 2.16 | | | 2.31 | | | 2.66 | | |
| **Time Taken (Hrs)**  **(199 individuals with 10000 SNPs 1000 replicates)** | | | | | 2.32 | | | 68.93 | | | 11.45 | | | 5.08 | | | 13.93 | | |

Chr: chromosome, r^2^: the proportion of phenotypic variance explained by each QTN; MSE: mean square error.
