# Supplementary material for: Iterative sure independence screening EM-Bayesian LASSO algorithm for multi-locus genome-wide association studies
Source: PLoS Comput Biol. 2017 Jan 31;13(1):e1005357. doi: 10.1371/journal.pcbi.1005357 (PMC5308866; doi:10.1371/journal.pcbi.1005357)
Supplement: S4 Table — (DOC) [file pcbi.1005357.s004.doc]

### S4 Table: Goodness of fit (AIC, BIC) for SNPs detected by four methods (ISIS EM-BLASSO (new), EMMA, FarmCPU and mrMLM), where a lower value indicates a better fit

| **Trait** | **ISIS EM-BLASSO** | | **EMMA** | | **FarmCPU** | | **mrMLM** | |
| --- | --- | --- | --- | --- | --- | --- | --- | --- |
|  | **AIC** | **BIC** | **AIC** | **BIC** | **AIC** | **BIC** | **AIC** | **BIC** |
| **LD** | 48.3534 | 98.2413 | 284.6000 | 289.7000 | 112.3335 | 146.6315 | -215.9000 | -67.5000 |
| **LDV** | -340.7140 | -284.4880 | -119.9000 | -104.5000 | -198.471 | -173.48 | -337.2000 | -260.4000 |
| **SD** | -342.7520 | -259.3860 | 113.1000 | 118.2000 | -54.5155 | -23.6395 | -367.9000 | -230.5000 |
| **0W** | -136.3320 | -69.1734 | 226.2000 | 226.2000 | 157.2206 | 174.7405 | -42.4000 | 21.5000 |
| **2W** | 9.5320 | 51.8663 | 216.4000 | 221.4000 | 70.8281 | 101.0669 | -140.6000 | -30.1000 |
| **4W** | -70.3529 | -31.4451 | 105.3000 | 114.9000 | 1.7465 | 26.7586 | -122.4000 | -55.5000 |

AIC: Akaike Information Criterion; BIC: Bayesian Information Criterion. LD: days to flowering under long days; LDV: days to flowering under long days with vernalization; SD: days to flowering under short days; 0W: days to flowering under long days for no vernalization; 2W: days to flowering under long days for 2 weeks vernalization; 4W: days to flowering under long days for 4 weeks vernalization.
