# Supplementary material for: Iterative sure independence screening EM-Bayesian LASSO algorithm for multi-locus genome-wide association studies
Source: PLoS Comput Biol. 2017 Jan 31;13(1):e1005357. doi: 10.1371/journal.pcbi.1005357 (PMC5308866; doi:10.1371/journal.pcbi.1005357)
Supplement: S5 Table — (DOC) [file pcbi.1005357.s005.doc]

### S5 Table: Genome-wide association studies for six flowering time traits in Arabidopsis thaliana using ISIS EM-BLASSO (new), EMMA, FarmCPU and mrMLM

| **Trait** | **Gene Name** | **Gene ID** | **Chr** | **SNP Position** | **ISIS EM-BLASSO** | | | **EMMA** | | | **FarmCPU** | | | **mrMLM** | | | | **Distance to Gene (bp)** | **References** |
| --- | --- | --- | --- | --- | --- | --- | --- | --- | --- | --- | --- | --- | --- | --- | --- | --- | --- | --- | --- |
|  |  |  |  |  | **r^2^** | **Effect** | **LOD** | **r^2^** | **Effect** | **P-value** | **r^2^** | **Effect** | **P-value** | **r^2^** | **Effect** | | **LOD** |  |  |
| **LD** | NTL | AT1G11570 | 1 | 3875883 | 1.65 | -0.11 | 3.95 |  |  |  |  |  |  |  |  | |  | -7397 | 1 |
|  | ARF1 | AT1G23490 | 1 | 8341601 | 1.83 | -0.10 | 5.68 | 7.05 | -0.20 | 2.83E-06 |  |  |  |  |  | |  | 2929 | 2 |
|  | SPA4 | AT1G53090 | 1 | 19802665 | 2.47 | -0.19 | 5.68 | 10.02 | -0.38 | 4.53E-06 |  |  |  |  |  | |  | -15763 | 3 |
|  | SPL4 | AT1G53160 | 1 | 19802665 | 2.47 | -0.19 | 5.68 |  |  |  |  |  |  |  |  | |  | 3754 | 4 |
|  | SVP | AT2G22540 | 2 | 9588685 | 2.70 | -0.14 | 4.97 | 24.59 | -0.41 | 2.78E-09 | 12.13 | -0.26 | 1.09E-12 |  |  | |  | 0 | 5 |
|  | VAR2 | AT2G30940 | 2 | 13143390 | 2.24 | -0.11 | 6.93 |  |  |  |  |  |  |  |  | |  | -25143 | 6 |
|  | ABCB19 | AT3G28860 | 3 | 10855475 | 2.99 | 0.15 | 6.40 |  |  |  |  |  |  | 1.56 | 0.07 | | 5.60 | -34563 | 7 |
|  | DOF4 | AT4G00940 | 4 | 387727 |  |  |  |  |  |  |  |  |  |  |  | |  | -15593 | 8 |
|  | ETC3 | AT4G01060 | 4 | 454542 |  |  |  | 8.22 | -0.22 | 3.17E-06 |  |  |  | 0.72 | -0.07 | | 4.70 | 5930 | 9 |
|  | ETC3 | AT4G01060 | 4 | 466800 | 1.43 | -0.09 | 4.41 |  |  |  | 6.44 | -0.19 | 3.84E-10 |  |  | |  | -5715 | 9 |
|  | EHD2 | AT4G05520 | 4 | 2819449 | 0.94 | -0.07 | 3.69 |  |  |  |  |  |  |  |  | |  | -11520 | 10 |
|  | ELP 6 | AT4G10090 | 4 | 6273257 | 2.34 | -0.13 | 7.36 |  |  |  |  |  |  |  |  | |  | 32111 | 11 |
|  | STK | AT4G09960 | 4 | 6273257 | 2.34 | -0.13 | 7.36 |  |  |  |  |  |  |  | |  |  | -32325 | 12 |
|  | APX3 | AT4G35000 | 4 | 16689198 | 1.67 | -0.10 | 4.81 |  |  |  |  |  |  |  | |  |  | -14427 | 13 |
|  | FLC | AT5G10140 | 5 | 3188328 | 1.85 | -0.11 | 5.11 | 9.44 | -0.26 | 8.82E-07 |  |  |  | 1.35 | | -0.10 | 9.48 | 8880 | 14 |
|  | LAC15 | AT5G48100 | 5 | 19469740 | 1.87 | -0.12 | 5.83 |  |  |  |  |  |  | 2.34 | | -0.08 | 6.12 | -19587 | 15 |
| **LDV** | MOS2 | AT1G33520 | 1 | 12179065 | 3.22 | -0.05 | 10.33 |  |  |  |  |  |  |  | |  |  | 20112 | 16 |
|  | FKF1 | AT1G68050 | 1 | 25508081 | 1.51 | 0.03 | 5.05 |  |  |  |  |  |  |  | |  |  | 595 | 17 |
|  | SVP | AT2G22540 | 2 | 9588685 | 4.49 | -0.06 | 5.04 |  |  |  |  |  |  | 2.98 | | -0.05 | 10.30 | 0 | 19 |
|  | CKB4 | AT2G44680 | 2 | 18446546 | 1.91 | -0.05 | 4.87 | 18.09 | -0.16 | 5.74E-08 | 17.80 | -0.16 | 8.54E-17 | 2.05 | | -0.05 | 5.75 | 18189 | 21 |
|  | HIRA | AT3G44530 | 3 | 16092326 | 2.41 | -0.06 | 4.30 |  |  |  |  |  |  | 1.67 | | -0.072 | 7.82 | -23262 | 23 |
|  | CESA1 | AT4G32410 | 4 | 15645842 | 1.26 | -0.04 | 3.74 |  |  |  | 8.16 | -0.10 | 1.73E-11 |  | |  |  | -823 | 26 |
|  | SMG7 | AT5G19400 | 5 | 6546259 | 3.06 | -0.06 | 6.66 |  |  |  |  |  |  |  | |  |  | 1244 | 27,28 |
|  | ATPI4K*3 | AT5G24240 | 5 | 8248050 | 2.45 | 0.06 | 5.89 |  |  |  |  |  |  |  | |  |  | 13900 | 29 |
|  | DOGI | AT5G45830 | 5 | 18599929 | 7.06 | -0.070 | 16.24 |  |  |  |  |  |  | 4.77 | | -0.06 | 7.25 | 8529 | 30 |
|  | HDA6 | AT5G63110 | 5 | 25290046 | 1.29 | -0.04 | 3.1465 | 10.56 | -0.13 | 2.35E-09 |  |  |  | 3.71 | | -0.06 | 5.71 | -25578 | 31 |
|  | GAMMA CAL1 | AT5G63510 | 5 | 25407568 |  |  |  |  |  |  | 12.01 | -0.12 | 4.51E-12 |  | |  |  | 16399 | 32 |
|  | AGL17 | AT2G22630 | 2 | 9588685 | 3.66 | -0.05 | 4.17 |  |  |  |  |  |  |  | |  |  | -29687 | 33 |
| **SD** | RAV1 | AT1G13260 | 1 | 4553530 | 2.32 | -0.07 | 13.61 |  |  |  |  |  |  |  | |  |  | 9788 | 34 |
|  | EXT3 | AT1G21310 | 1 | 7448446 | 0.37 | -0.02 | 3.64 |  |  |  |  |  |  |  | |  |  | -4790 | 35 |
|  | ATABA2 | AT1G52340 | 1 | 19483362 | 0.10 | 0.01 | 3.19 |  |  |  |  |  |  | 2.54 | | 0.042 | 6.01 | -6432 | 38 |
|  | AT2G12400 | AT2G12400 | 2 | 4999298 | 0.96 | -0.04 | 8.48 |  |  |  |  |  |  |  | |  |  | -5645 | 39 |
|  | PS2 | AT2G29900 | 2 | 12757930 | 1.16 | -0.07 | 9.93 |  |  |  | 4.76 | -0.13 | 1.06E-13 |  | |  |  | 6805 | 40 |
|  | PAP13 | AT2G32770 | 2 | 13912889 | 3.45 | -0.10 | 21.88 |  |  |  |  |  |  |  | |  |  | 14612 | 41 |
|  | SPA1 | AT2G46340 | 2 | 19044616 | 0.63 | -0.04 | 5.65 |  |  |  |  |  |  |  | |  |  | 17203 | 42 |
|  | PDF1 | AT3G25800 | 3 | 9437458 | 0.24 | 0.03 | 4.16 |  |  |  |  |  |  |  | |  |  | 11414 | 43 |
|  | CDG1 | AT3G26940 | 3 | 9926056 | 0.65 | -0.03 | 6.38 |  |  |  |  |  |  |  | |  |  | -10526 | 44 |
|  | ABCB19 | AT3G28860 | 3 | 10855475 | 3.99 | 0.09 | 18.50 |  |  |  | 4.08 | 0.09 | 3.96E-09 |  | |  |  | -14563 | 7 |
|  | ATJ3 | AT3G44110 | 3 | 15833892 | 0.40 | -0.03 | 4.35 |  |  |  |  |  |  |  | |  |  | -34902 | 45 |
|  | ELP6 | AT4G10090 | 4 | 6272658 | 0.42 | -0.03 | 4.45 |  |  |  |  |  |  |  |  | |  | -32710 | 46 |
|  | CKL11 | AT4G14340 | 4 | 8228311 | 0.24 | -0.03 | 3.90 |  |  |  |  |  |  |  |  | |  | -19975 | 47 |
|  | RALFL33 | AT4G15800 | 4 | 8950037 | 1.14 | 0.07 | 7.70 |  |  |  | 5.62 | 0.13 | 2.04E-09 |  |  | |  | -34821 | 48 |
|  | EBS | AT4G22140 | 4 | 11707246 | 1.05 | 0.06 | 9.07 |  |  |  |  |  |  |  |  | |  | -20480 | 49 |
|  | CESA1 | AT4G32410 | 4 | 15645842 | 1.95 | -0.07 | 13.21 |  |  |  |  |  |  |  |  | |  | -823 | 26 |
|  | sim to VRN1 | AT4G33280 | 4 | 16083817 | 2.56 | -0.08 | 12.19 |  |  |  | 7.72 | -0.14 | 2.41E-15 |  |  | |  | 34462 | 50 |
|  | CYCLIN 1 | AT4G37490 | 4 | 17632478 | 1.75 | -0.05 | 11.87 |  |  |  |  |  |  |  |  | |  | 8193 | 51 |
|  | STN8 | AT5G01920 | 5 | 338648 | 0.44 | -0.03 | 4.33 |  |  |  |  |  |  |  |  | |  | -20459 | 52 |
|  | ATXR5 | AT5G09790 | 5 | 3051259 | 0.78 | -0.04 | 6.14 |  |  |  |  |  |  | 4.50 | -0.04 | | 6.23 | 10215 | 53 |
|  | NAC089 | AT5G22290 | 5 | 7379995 | 0.54 | -0.05 | 5.43 |  |  |  |  |  |  |  |  | |  | 2351 | 54 |
|  | DOGI | AT5G45830 | 5 | 18627646 | 2.79 | -0.07 | 14.09 |  |  |  |  |  |  |  |  | |  | 36246 | 30 |
|  | GAMMA CAL1 | AT5G63510 | 5 | 25378781 | 0.79 | -0.05 | 6.40 |  |  |  |  |  |  |  |  | |  | -45186 | 55 |
| **0W** | FKF1 | AT1G68050 | 1 | 6371569 | 0.42 | -0.05 | 3.05 |  |  |  |  |  |  |  |  | |  | 595 | 17 |
|  | SPDS1 | AT1G23730 | 1 | 8392979 | 0.57 | 0.06 | 4.39 |  |  |  |  |  |  |  |  | |  | 27286 | 56 |
|  | SPDS1 | AT1G23820 | 1 | 8417196 | 1.23 | 0.07 | 7.74 |  |  |  |  |  |  |  |  | |  | -3069 | 56 |
|  | GRP3S | AT2G05380 | 2 | 1921172 | 1.73 | -0.10 | 6.22 |  |  |  |  |  |  |  |  | |  | -45644 | 57 |
|  | ERD15 | AT2G41430 | 2 | 17285257 | 0.77 | -0.05 | 4.56 |  |  |  |  |  |  |  |  | |  | 14715 | 58 |
|  | ADL4 | AT3G60190 | 3 | 22255698 | 0.44 | -0.05 | 3.18 |  |  |  |  |  |  |  |  | |  | 7865 | 59 |
|  | ETC3 | AT4G01060 | 4 | 429928 | 6.70 | -0.15 | 24.23 |  |  |  |  |  |  | 3.53 | -0.08 | | 3.54 | -30544 | 9 |
|  | GAI | AT4G02780 | 4 | 1228186 | 2.17 | -0.09 | 8.25 |  |  |  |  |  |  |  |  | |  | -9581 | 60 |
|  | BRN1 | AT4G03110 | 4 | 1345545 | 2.26 | -0.09 | 9.89 |  |  |  |  |  |  |  |  | |  | -30761 | 61 |
|  | NRGA1 | AT4G05590 | 4 | 2903746 | 1.25 | -0.07 | 8.73 |  |  |  |  |  |  |  |  | |  | -3319 | 62 |
|  | NIP5;1 | AT4G10380 | 4 | 6415030 | 0.30 | 0.04 | 3.36 |  |  |  |  |  |  |  |  | |  | -16206 | 63 |
|  | RAP74 | AT4G12610 | 4 | 7459086 | 1.30 | 0.09 | 6.32 |  |  |  |  |  |  |  |  | |  | 768 | 64 |
|  | AGL19 | AT4G22950 | 4 | 12048235 | 1.33 | -0.07 | 7.45 |  |  |  |  |  |  |  |  | |  | 20814 | 65 |
|  | CESA1 | AT4G32410 | 4 | 15645842 | 1.97 | -0.11 | 7.70 |  |  |  |  |  |  |  |  | |  | -823 | 26 |
|  | HAM4 | AT4G36710 | 4 | 17325234 | 0.76 | 0.0484 | 5.43 |  |  |  |  |  |  |  |  | |  | 17591 | 66 |
|  | ATXR5 | AT5G09790 | 5 | 3037582 | 0.45 | 0.04 | 4.48 |  |  |  |  |  |  |  |  | |  | -1396 | 53 |
|  | ROXY2 | AT5G14070 | 5 | 4867634 | 1.01 | -0.07 | 5.01 |  |  |  |  |  |  |  |  | |  | -8881 | 67 |
|  | SPL7 | AT5G18830 | 5 | 6289819 | 0.53 | -0.04 | 4.46 |  |  |  |  |  |  |  |  | |  | 9138 | 68 |
|  | DGR2 | AT5G25460 | 5 | 8844702 | 2.46 | -0.09 | 8.34 |  |  |  |  |  |  |  |  | |  | -18661 | 69 |
|  | AGL26 | AT5G26880 | 5 | 9416556 | 0.97 | -0.06 | 4.26 |  |  |  |  |  |  |  |  | |  | -40481 | 70 |
|  | DOGI | AT5G45830 | 5 | 18599929 | 6.26 | -0.15 | 20.93 |  |  |  |  |  |  | 3.56 | -0.15 | | 11.87 | 8529 | 30 |
| **2W** | CLPP6 | AT1G11750 | 1 | 3978063 |  |  |  | 15.38 | -0.33 | 5.16E-07 | 5.94 | -0.20 | 8.28E-12 | 4.56 | -0.16 | | 13.38 | 8186 | 71 |
|  | DD46 | AT1G22015 | 1 | 7758148 | 2.44 | -0.13 | 5.66 |  |  |  |  |  |  |  |  | |  | 4629 | 72 |
|  | LPAT3 | AT1G51260 | 1 | 19008023 | 1.76 | -0.10 | 3.8131 |  |  |  |  |  |  |  |  | |  | 2382 | 73 |
|  | PAP13 | AT2G32770 | 2 | 13912889 | 4.12 | -0.17 | 5.62 | 15.06 | -0.34 | 4.91E-08 | 9.96 | -0.28 | 3.23E-11 | 3.56 | -0.110 | | 6.98 | 14612 | 41 |
|  | JAL35 | AT3G16470 | 3 | 5579959 | 2.33 | -0.15 | 5.31 |  |  |  | 7.84 | -0.27 | 1.09E-12 | 2.45 | -0.19 | | 13.92 | -15970 | 74 |
|  | ABCB19 | AT3G28860 | 3 | 10855475 | 1.77 | 0.10 | 3.12 |  |  |  |  |  |  |  |  | |  | -14563 | 7 |
|  | ATRBL11 | AT3G58460 | 3 | 21613189 | 1.10 | -0.09 | 3.06 |  |  |  |  |  |  |  |  | |  | -5206 | 75 |
|  | NGA2 | AT3G61970 | 3 | 22949227 | 1.89 | 0.09 | 4.43 |  |  |  |  |  |  |  |  | |  | -2236 | 76 |
|  | ETC3 | AT4G01060 | 4 | 454542 | 2.42 | -0.11 | 5.62 | 9.95 | -0.21 | 8.07E-07 | 3.50 | -0.12 | 7.08E-08 | 1.72 | -0.09 | | 7.18 | -5930 | 9 |
|  | SPL7 | AT5G18830 | 5 | 6289819 | 1.24 | -0.07 | 3.310 |  |  |  |  |  |  | 1.75 | -0.086 | | 6.39 | 9138 | 68 |
| **4W** | ELP | AT1G05850 | 1 | 1771680 | 2.00 | -0.11 | 6.94 |  |  |  |  |  |  |  |  | |  | 2985 | 77 |
|  | CLPP6 | AT1G11750 | 1 | 3978063 | 6.11 | -0.18 | 10.38 | 23.44 | -0.35 | 1.61E-07 | 11.07 | -0.24 | 3.31E-14 | 2.56 | -0.21 | | 15.82 | 8186 | 71 |
|  | ARF1 | AT1G23490 | 1 | 8341601 | 1.28 | -0.07 | 4.31 |  |  |  |  |  |  | 1.34 | -0.07 | | 4.65 | 2929 | 2 |
|  | SVP | AT2G22630 | 2 | 9588685 |  |  |  | 30.03 | -0.37 | 1.97E-08 |  |  |  |  |  | |  | -29687 | 5 |
|  | WRKY25 | AT2G30250 | 2 | 12916879 | 1.91 | -0.09 | 6.44 |  |  |  |  |  |  |  |  | |  | 11681 | 79 |
|  | GSTF9 | AT2G30860 | 2 | 13143876 | 2.67 | -0.12 | 7.52 |  |  |  |  |  |  |  |  | |  | 3631 | 80 |
|  | CCT1 | AT2G32260 | 2 | 13701496 | 0.71 | -0.05 | 3.42 |  |  |  |  |  |  |  |  | |  | 855 | 81 |
|  | JAL35 | AT3G16470 | 3 | 5579959 | 0.95 | -0.08 | 3.0 |  |  |  | 4.21 | -0.17 | 2.48E-11 | 1.35 | -0.17 | | 14.64 | -15970 | 76 |
|  | ABCB19 | AT3G28860 | 3 | 10855475 | 3.08 | 0.12 | 7.67 | 7.63 | 0.19 | 6.84E-07 |  |  |  |  |  | |  | -14563 | 7 |
|  | SMG7 | AT5G19400 | 5 | 6525973 | 5.21 | -0.19 | 11.00 |  |  |  |  |  |  |  |  | |  | -14030 | 27, 28 |
|  | DOGI | AT5G45830 | 5 | 18607728 | 6.86 | -0.20 | 14.65 |  |  |  |  |  |  | 4.69 | -0.16 | | 10.09 | 16328 | 30 |
|  | CKB1 | AT5G47080 | 5 | 19139239 | 0.97 | 0.06 | 3.64 |  |  |  |  |  |  |  |  | |  | 12586 | 82 |

LD: days to flowering under long days; LDV: days to flowering under long days with vernalization; SD: days to flowering under short days; 0W: days to flowering under long days for no vernalization; 2W: days to flowering under long days for 2 weeks vernalization; 4W: days to flowering under long days for 4 weeks vernalization. All the references in this table are listed in below.

# References cited in Table S5

1. Kim SG, [Park](http://www.tandfonline.com/author/Park%2C+C) CM: **Membrane-Mediated Salt Stress Signaling in Flowering Time Control.** *Plant Signaling & Behavior* 2007; **2(6)**, 517–518.
2. Ellis CM, Nagpal P, Young JC, Hagen G, Guilfoyle TJ, Reed JW: **AUXIN RESPONSE FACTOR1 and AUXIN RESPONSE FACTOR2 regulate senescence and floral organ abscission in Arabidopsis thaliana.** *Development* 2005; **132**, 4563-74.doi: 10.1242/dev.02012.
3. Imaizumi T, [Kay](https://scholar.google.com/citations?user=55sPPYMAAAAJ&hl=en&oi=sra) SA: [**Photoperiodic control of flowering: not only by coincidence**](http://www.sciencedirect.com/science/article/pii/S1360138506002500)**.** *Trends in plant science* 2006; [**11(11**](http://www.sciencedirect.com/science/journal/13601385/11/11)**)**, 550–558.
4. Jung JH, Ju Y, Seo PJ, Lee JH, Park CM: **The SOC1-SPL module integrates photoperiod and gibberellic acid signals to control flowering time in Arabidopsis**. *The Plant Journal* 2012; **69(4)**, 577–588. DOI: 10.1111/j.1365-313X.2011.04813.x.
5. [Lee](http://genesdev.cshlp.org/search?author1=Jeong+Hwan+Lee&sortspec=date&submit=Submit) JH,  [Yoo](http://genesdev.cshlp.org/search?author1=Seong+Jeon+Yoo&sortspec=date&submit=Submit) SJ, [Park](http://genesdev.cshlp.org/search?author1=Soo+Hyun+Park&sortspec=date&submit=Submit) SH, [Hwang](http://genesdev.cshlp.org/search?author1=Ildoo+Hwang&sortspec=date&submit=Submit) I, [Lee](http://genesdev.cshlp.org/search?author1=Jong+Seob+Lee&sortspec=date&submit=Submit) JS, and [Ahn](http://genesdev.cshlp.org/search?author1=Ji+Hoon+Ahn&sortspec=date&submit=Submit) JH: **Role of SVP in the control of flowering time by ambient temperature in Arabidopsis.** *Genes & Dev* 2007; **21**, 397-402.
6. Rosloski SM, Singh A, Jali SS, Balasubramanian S, Weigel D, Grbic V: [**Functional analysis of splice variant expression of MADS AFFECTING FLOWERING 2 of Arabidopsis thaliana**](https://www.ncbi.nlm.nih.gov/pubmed/23111501). [*Plant Molecular Biology*](http://link.springer.com/journal/11103) 2013; **81(1-2)**, 57-69. doi: 10.1007/s11103-012-9982-2
7. Kavai-ool, UN:  **Genetic regulation of polar auxin transport and its role in control of shoot morphogenesis**. [*Biology Bulletin Reviews*](http://link.springer.com/journal/13335)2011; 1(6), 517. doi:10.1134/S207908641106003X
8. Gao J, Zhang Y, Zhang C, Qi F, Li X, Mu S, Peng Z: **Characterization of the Floral Transcriptome of Moso Bamboo (Phyllostachys edulis) at Different Flowering Developmental Stages by Transcriptome Sequencing and RNA-Seq Analysis**. *PLoS ONE* 2014; **9(6)**: e98910. doi:10.1371/journal.pone.0098910.
9. Brachi B, Faure N, Horton M, Flahauw E, Vazquez A, Nordborg M,  [Bergelson J](https://www.ncbi.nlm.nih.gov/pubmed/?term=Bergelson J%5BAuthor%5D&cauthor=true&cauthor_uid=20463887), [Cuguen J](https://www.ncbi.nlm.nih.gov/pubmed/?term=Cuguen J%5BAuthor%5D&cauthor=true&cauthor_uid=20463887), [Roux F](https://www.ncbi.nlm.nih.gov/pubmed/?term=Roux F%5BAuthor%5D&cauthor=true&cauthor_uid=20463887): **Linkage and Association Mapping of Arabidopsis thaliana Flowering Time in Nature**. *PLoS Genet*. 2010; **6(5)**: e1000940. doi:10.1371/journal.pgen.1000940.
10. [Wei X](https://www.ncbi.nlm.nih.gov/pubmed/?term=Wei X%5BAuthor%5D&cauthor=true&cauthor_uid=20566706), [Xu J](https://www.ncbi.nlm.nih.gov/pubmed/?term=Xu J%5BAuthor%5D&cauthor=true&cauthor_uid=20566706), [Guo H](https://www.ncbi.nlm.nih.gov/pubmed/?term=Guo H%5BAuthor%5D&cauthor=true&cauthor_uid=20566706), [Jiang L](https://www.ncbi.nlm.nih.gov/pubmed/?term=Jiang L%5BAuthor%5D&cauthor=true&cauthor_uid=20566706), [Chen S](https://www.ncbi.nlm.nih.gov/pubmed/?term=Chen S%5BAuthor%5D&cauthor=true&cauthor_uid=20566706), [Yu C](https://www.ncbi.nlm.nih.gov/pubmed/?term=Yu C%5BAuthor%5D&cauthor=true&cauthor_uid=20566706), [Zhou Z](https://www.ncbi.nlm.nih.gov/pubmed/?term=Zhou Z%5BAuthor%5D&cauthor=true&cauthor_uid=20566706), [Hu P](https://www.ncbi.nlm.nih.gov/pubmed/?term=Hu P%5BAuthor%5D&cauthor=true&cauthor_uid=20566706), [Zhai H](https://www.ncbi.nlm.nih.gov/pubmed/?term=Zhai H%5BAuthor%5D&cauthor=true&cauthor_uid=20566706), [Wan J](https://www.ncbi.nlm.nih.gov/pubmed/?term=Wan J%5BAuthor%5D&cauthor=true&cauthor_uid=20566706): **DTH8 Suppresses Flowering in Rice, Influencing Plant Height and Yield Potential simultaneously**. *Plant Physiology* 2010; **153(4)**, 1747-58.  doi: 10.1104/pp.110.156943
11. Yuan L, Liu X, Luo M, Yang S, and Wu K: [**Involvement of histone modifications in plant abiotic stress responses**](http://onlinelibrary.wiley.com/doi/10.1111/jipb.12060/full). *Journal of Integrative Plant Biology* 2013; [**55(10)**](http://onlinelibrary.wiley.com/doi/10.1111/jipb.v55.10/issuetoc) 892–901.  doi: 10.1111/jipb.12060
12. Xu J, Zhong X, Zhang Q, Li H: **Overexpression of the GmGAL2 Gene Accelerates Flowering in Arabidopsis.** [*Plant Molecular Biology Report*](http://link.springer.com/journal/11105) 2010; **28(4)**, 704-711. doi:  [10.1007/s11105-010-0201-5](https://dx.doi.org/10.1007%2Fs11105-010-0201-5)
13. Becker B, Holtgrefe S, Jung S, Wunrau C, Kandlbinder A, Baier M, Dietz KJ,  Backhausen JE, Scheibe R: **Influence of the photoperiod on redox regulation and stress responses in Arabidopsis thaliana L. (Heynh.)plants under long- and short-day conditions**. [*Planta*](http://link.springer.com/journal/425) 2006; 224(2), 380-93.
14. Caicedo AL, Stinchcombe JR, Olsen KM, Schmitt J, Purugganan MD: **Epistatic interaction between Arabidopsis FRI and FLC flowering time genes generates a latitudinal cline in a life history trait**. *PNAS*. 2004; **101**, 15670–5.
15. Cai X, [Davis](http://jxb.oxfordjournals.org/search?author1=Elizabeth+J.+Davis&sortspec=date&submit=Submit) EJ, [Ballif](http://jxb.oxfordjournals.org/search?author1=Jenny+Ballif&sortspec=date&submit=Submit) J, [Liang](http://jxb.oxfordjournals.org/search?author1=Mingxiang+Liang&sortspec=date&submit=Submit) M,  [Bushman](http://jxb.oxfordjournals.org/search?author1=Emily+Bushman&sortspec=date&submit=Submit) E, [Haroldsen](http://jxb.oxfordjournals.org/search?author1=Victor+Haroldsen&sortspec=date&submit=Submit) V, [Torabinejad](http://jxb.oxfordjournals.org/search?author1=Javad+Torabinejad&sortspec=date&submit=Submit) J, and [Wu](http://jxb.oxfordjournals.org/search?author1=Yajun+Wu&sortspec=date&submit=Submit) Y: **Mutant identification and characterization of the laccase gene family in Arabidopsis**. *J. Exp. Bot.* 2006; **57 (11)**, 2563-9.
16. [Palma K](https://www.ncbi.nlm.nih.gov/pubmed/?term=Palma K%5BAuthor%5D&cauthor=true&cauthor_uid=17575050), [Zhao Q](https://www.ncbi.nlm.nih.gov/pubmed/?term=Zhao Q%5BAuthor%5D&cauthor=true&cauthor_uid=17575050), [Cheng YT](https://www.ncbi.nlm.nih.gov/pubmed/?term=Cheng YT%5BAuthor%5D&cauthor=true&cauthor_uid=17575050), [Bi D](https://www.ncbi.nlm.nih.gov/pubmed/?term=Bi D%5BAuthor%5D&cauthor=true&cauthor_uid=17575050), [Monaghan J](https://www.ncbi.nlm.nih.gov/pubmed/?term=Monaghan J%5BAuthor%5D&cauthor=true&cauthor_uid=17575050), [Cheng W](https://www.ncbi.nlm.nih.gov/pubmed/?term=Cheng W%5BAuthor%5D&cauthor=true&cauthor_uid=17575050), [Zhang Y](https://www.ncbi.nlm.nih.gov/pubmed/?term=Zhang Y%5BAuthor%5D&cauthor=true&cauthor_uid=17575050), [Li X](https://www.ncbi.nlm.nih.gov/pubmed/?term=Li X%5BAuthor%5D&cauthor=true&cauthor_uid=17575050): **Regulation of plant innate immunity by three proteins in a complex conserved across the plant and animal kingdoms**. *Genes Dev****.*** 21(12),1484-93.
17. [Imaizumi T](https://www.ncbi.nlm.nih.gov/pubmed/?term=Imaizumi T%5BAuthor%5D&cauthor=true&cauthor_uid=16002617), [Schultz TF](https://www.ncbi.nlm.nih.gov/pubmed/?term=Schultz TF%5BAuthor%5D&cauthor=true&cauthor_uid=16002617), [Harmon FG](https://www.ncbi.nlm.nih.gov/pubmed/?term=Harmon FG%5BAuthor%5D&cauthor=true&cauthor_uid=16002617), [Ho LA](https://www.ncbi.nlm.nih.gov/pubmed/?term=Ho LA%5BAuthor%5D&cauthor=true&cauthor_uid=16002617), [Kay SA](https://www.ncbi.nlm.nih.gov/pubmed/?term=Kay SA%5BAuthor%5D&cauthor=true&cauthor_uid=16002617): [FKF1 F-box protein mediates cyclic degradation of a repressor of CONSTANS in Arabidopsis](http://www.sciencemag.org/content/309/5732/293.short). *Science* 2005; 309(5732), 293-7.
18. [Zhang H](https://www.ncbi.nlm.nih.gov/pubmed/?term=Zhang H%5BAuthor%5D&cauthor=true&cauthor_uid=12207655), [van Nocker S](https://www.ncbi.nlm.nih.gov/pubmed/?term=van Nocker S%5BAuthor%5D&cauthor=true&cauthor_uid=12207655): **The VERNALIZATION INDEPENDENCE 4 gene encodes a novel regulator of FLOWERING LOCUS C.** *The Plant J*. 2002; **31(5),** 663-73.
19. Lee JH, Yoo SJ, Park SH, Hwang I, Lee JS, Ahn JH: **Role of SVP in the control of flowering time by ambient temperature in Arabidopsis.** *Genes Dev.* 2007; **21(4)**, 397-402.
20. [Alvarez-Buylla ER](https://www.ncbi.nlm.nih.gov/pubmed/?term=Alvarez-Buylla ER%5BAuthor%5D&cauthor=true&cauthor_uid=11115127), [Liljegren SJ](https://www.ncbi.nlm.nih.gov/pubmed/?term=Liljegren SJ%5BAuthor%5D&cauthor=true&cauthor_uid=11115127), [Pelaz S](https://www.ncbi.nlm.nih.gov/pubmed/?term=Pelaz S%5BAuthor%5D&cauthor=true&cauthor_uid=11115127), [Gold SE](https://www.ncbi.nlm.nih.gov/pubmed/?term=Gold SE%5BAuthor%5D&cauthor=true&cauthor_uid=11115127), [Burgeff C](https://www.ncbi.nlm.nih.gov/pubmed/?term=Burgeff C%5BAuthor%5D&cauthor=true&cauthor_uid=11115127), [Ditta GS](https://www.ncbi.nlm.nih.gov/pubmed/?term=Ditta GS%5BAuthor%5D&cauthor=true&cauthor_uid=11115127), [*et al*](https://www.ncbi.nlm.nih.gov/pubmed/?term=Vergara-Silva F%5BAuthor%5D&cauthor=true&cauthor_uid=11115127).: [**MADS‐box gene evolution beyond flowers: expression in pollen, endosperm, guard cells, roots and trichomes**](http://onlinelibrary.wiley.com/doi/10.1111/j.1365-313X.2000.00891.x/pdf)**.**  *The Plant J.* 2009; **24(4)**, 457-66.
21. [Mulekar JJ](https://www.ncbi.nlm.nih.gov/pubmed/?term=Mulekar JJ%5BAuthor%5D&cauthor=true&cauthor_uid=22353866), [Huq E](https://www.ncbi.nlm.nih.gov/pubmed/?term=Huq E%5BAuthor%5D&cauthor=true&cauthor_uid=22353866): **Does CK2 affect flowering time by modulating the autonomous pathway in Arabidopsis?** [*Plant Signal Behav.*](https://www.ncbi.nlm.nih.gov/pubmed/?term=Does+CK2+affect+flowering+time+by+modulating+the+autonomous+pathway+in+Arabidopsis%3F) 2012; **7(2)**, 292-94. doi: 10.4161/psb.18883.
22. [Kaldis A](https://www.ncbi.nlm.nih.gov/pubmed/?term=Kaldis A%5BAuthor%5D&cauthor=true&cauthor_uid=21193996), [Tsementzi D](https://www.ncbi.nlm.nih.gov/pubmed/?term=Tsementzi D%5BAuthor%5D&cauthor=true&cauthor_uid=21193996), [Tanriverdi O](https://www.ncbi.nlm.nih.gov/pubmed/?term=Tanriverdi O%5BAuthor%5D&cauthor=true&cauthor_uid=21193996), [Vlachonasios KE](https://www.ncbi.nlm.nih.gov/pubmed/?term=Vlachonasios KE%5BAuthor%5D&cauthor=true&cauthor_uid=21193996): [**Arabidopsis thaliana transcriptional co-activators ADA2b and SGF29a are implicated in salt stress responses**](http://link.springer.com/article/10.1007/s00425-010-1337-0)**.** [*Planta*](https://www.ncbi.nlm.nih.gov/pubmed/21193996) 2011; **233(4)**, 749-62.
23. [Zhu](http://www.sciencedirect.com/science/article/pii/S1874939911001350) Y, [Dong](http://www.sciencedirect.com/science/article/pii/S1874939911001350) A,  [Shen](http://www.sciencedirect.com/science/article/pii/S1874939911001350) WH: **Histone variants and chromatin assembly in plant abiotic stress responses**. [*Biochimica et Biophysica Acta (BBA) - Gene Regulatory Mechanisms*](http://www.sciencedirect.com/science/journal/18749399) 2012;[**1819-3(4**](http://www.sciencedirect.com/science/journal/18749399/1819/3)**)**, 343–48. [dx.doi.org/10.1016/j.bbagrm.2011.07.012](http://dx.doi.org/10.1016/j.bbagrm.2011.07.012)
24. [Deng W](https://www.ncbi.nlm.nih.gov/pubmed/?term=Deng W%5BAuthor%5D&cauthor=true&cauthor_uid=21464308), [Ying H](https://www.ncbi.nlm.nih.gov/pubmed/?term=Ying H%5BAuthor%5D&cauthor=true&cauthor_uid=21464308), [Helliwell CA](https://www.ncbi.nlm.nih.gov/pubmed/?term=Helliwell CA%5BAuthor%5D&cauthor=true&cauthor_uid=21464308), [Taylor JM](https://www.ncbi.nlm.nih.gov/pubmed/?term=Taylor JM%5BAuthor%5D&cauthor=true&cauthor_uid=21464308), [Peacock WJ](https://www.ncbi.nlm.nih.gov/pubmed/?term=Peacock WJ%5BAuthor%5D&cauthor=true&cauthor_uid=21464308), [Dennis ES](https://www.ncbi.nlm.nih.gov/pubmed/?term=Dennis ES%5BAuthor%5D&cauthor=true&cauthor_uid=21464308): [**FLOWERING LOCUS C (FLC) regulates development pathways throughout the life cycle of Arabidopsis**](http://www.pnas.org/content/108/16/6680.short)**.** [*Proc Natl Acad Sci U S A.*](https://www.ncbi.nlm.nih.gov/pubmed/?term=FLOWERING+LOCUS+C+(FLC)+regulates+development+pathways+throughout+the+life+cycle+of+Arabidopsis.) 2011; **108(16)**, 6680-5. doi: 10.1073/pnas.1103175108.
25. [Valverde](http://www.sciencemag.org/search?author1=Federico+Valverde&sortspec=date&submit=Submit) [F, Mouradov](http://www.sciencemag.org/search?author1=Aidyn+Mouradov&sortspec=date&submit=Submit) [A, Soppe](http://www.sciencemag.org/search?author1=Wim+Soppe&sortspec=date&submit=Submit) [W, Ravenscroft](http://www.sciencemag.org/search?author1=Dean+Ravenscroft&sortspec=date&submit=Submit) [D, Samach](http://www.sciencemag.org/search?author1=Alon+Samach&sortspec=date&submit=Submit) A, [Coupland](http://www.sciencemag.org/search?author1=George+Coupland&sortspec=date&submit=Submit) G: **Photoreceptor Regulation of CONSTANS Protein in Photoperiodic Flowering.** *Science* 2004; **303(5660)**, 1003-6. doi:[10.1126/science.1091761](https://dx.doi.org/10.1126/science.1091761)
26. Hamann T, Osborne E, Youngs LH, Misson J, Nussaume L, Somerville C: [Global expression analysis of CESA and CSL genes in Arabidopsis](http://link.springer.com/article/10.1023/B:CELL.0000046340.99925.57)**.** [*Cellulose*](http://link.springer.com/journal/10570)2004; 11(3), 279-86. doi:10.1023/B:CELL.0000046340.99925.57
27. [Riehs N](https://www.ncbi.nlm.nih.gov/pubmed/?term=Riehs N%5BAuthor%5D&cauthor=true&cauthor_uid=18544632), [Akimcheva S](https://www.ncbi.nlm.nih.gov/pubmed/?term=Akimcheva S%5BAuthor%5D&cauthor=true&cauthor_uid=18544632), [Puizina J](https://www.ncbi.nlm.nih.gov/pubmed/?term=Puizina J%5BAuthor%5D&cauthor=true&cauthor_uid=18544632), [Bulankova P](https://www.ncbi.nlm.nih.gov/pubmed/?term=Bulankova P%5BAuthor%5D&cauthor=true&cauthor_uid=18544632), [Idol RA](https://www.ncbi.nlm.nih.gov/pubmed/?term=Idol RA%5BAuthor%5D&cauthor=true&cauthor_uid=18544632), [Siroky J](https://www.ncbi.nlm.nih.gov/pubmed/?term=Siroky J%5BAuthor%5D&cauthor=true&cauthor_uid=18544632), [Schleiffer A](https://www.ncbi.nlm.nih.gov/pubmed/?term=Schleiffer A%5BAuthor%5D&cauthor=true&cauthor_uid=18544632), [Schweizer D](https://www.ncbi.nlm.nih.gov/pubmed/?term=Schweizer D%5BAuthor%5D&cauthor=true&cauthor_uid=18544632), [Shippen DE](https://www.ncbi.nlm.nih.gov/pubmed/?term=Shippen DE%5BAuthor%5D&cauthor=true&cauthor_uid=18544632), [Riha K](https://www.ncbi.nlm.nih.gov/pubmed/?term=Riha K%5BAuthor%5D&cauthor=true&cauthor_uid=18544632): **Arabidopsis SMG7 protein is required for exit from meiosis.** [*J Cell Sci.*](https://www.ncbi.nlm.nih.gov/pubmed/?term=Arabidopsis+SMG7+protein+is+required+for+exit+from+meiosis.)2008; **121(Pt 13)**, 2208-16; doi: 10.1242/jcs.027862.
28. [Vicente-Crespo M](https://www.ncbi.nlm.nih.gov/pubmed/?term=Vicente-Crespo M%5BAuthor%5D&cauthor=true&cauthor_uid=21118115)^1^, [Palacios IM](https://www.ncbi.nlm.nih.gov/pubmed/?term=Palacios IM%5BAuthor%5D&cauthor=true&cauthor_uid=21118115): **Nonsense-mediated mRNA decay and development: shoot the messenger to survive?** [*Biochem Soc Trans.* 2010; **38(6)**, 1500–5](http://www.ncbi.nlm.nih.gov/entrez/eutils/elink.fcgi?dbfrom=pubmed&retmode=ref&cmd=prlinks&id=21118115). doi: 10.1042/BST0381500.
29. [Akhter S](https://www.ncbi.nlm.nih.gov/pubmed/?term=Akhter S%5BAuthor%5D&cauthor=true&cauthor_uid=25879253), [Uddin MN](https://www.ncbi.nlm.nih.gov/pubmed/?term=Uddin MN%5BAuthor%5D&cauthor=true&cauthor_uid=25879253), [Jeong IS](https://www.ncbi.nlm.nih.gov/pubmed/?term=Jeong IS%5BAuthor%5D&cauthor=true&cauthor_uid=25879253), [Kim DW](https://www.ncbi.nlm.nih.gov/pubmed/?term=Kim DW%5BAuthor%5D&cauthor=true&cauthor_uid=25879253), [Liu XM](https://www.ncbi.nlm.nih.gov/pubmed/?term=Liu XM%5BAuthor%5D&cauthor=true&cauthor_uid=25879253), [Bahk JD](https://www.ncbi.nlm.nih.gov/pubmed/?term=Bahk JD%5BAuthor%5D&cauthor=true&cauthor_uid=25879253). **Role of Arabidopsis AtPI4Kγ3, a type II phosphoinositide 4-kinase, in abiotic stress responses and floral transition.** *Plant Biotechnol. J.* 2016; **14 (1)**: 215-30. doi: 10.1111/pbi.12376.
30. [Bentsink](http://www.pnas.org/search?author1=Leónie+Bentsink&sortspec=date&submit=Submit) L, [Jowett](http://www.pnas.org/search?author1=Jemma+Jowett&sortspec=date&submit=Submit) J, [Hanhart](http://www.pnas.org/search?author1=Corrie+J.+Hanhart&sortspec=date&submit=Submit) CJ, and [Koornneef](http://www.pnas.org/search?author1=Maarten+Koornneef&sortspec=date&submit=Submit) M: **Cloning of DOG1, a quantitative trait locus controlling seed dormancy in Arabidopsis.** [*Proc Natl Acad Sci U S A*](https://www.ncbi.nlm.nih.gov/pmc/articles/PMC1636575/)*.* 2006; **103(45)**: 17042–17047. doi:  [10.1073/pnas.0607877103](https://dx.doi.org/10.1073%2Fpnas.0607877103)
31. [Probst AV](https://www.ncbi.nlm.nih.gov/pubmed/?term=Probst AV%5BAuthor%5D&cauthor=true&cauthor_uid=15037732), [Fagard M](https://www.ncbi.nlm.nih.gov/pubmed/?term=Fagard M%5BAuthor%5D&cauthor=true&cauthor_uid=15037732), [Proux F](https://www.ncbi.nlm.nih.gov/pubmed/?term=Proux F%5BAuthor%5D&cauthor=true&cauthor_uid=15037732), [Mourrain P](https://www.ncbi.nlm.nih.gov/pubmed/?term=Mourrain P%5BAuthor%5D&cauthor=true&cauthor_uid=15037732), [Boutet S](https://www.ncbi.nlm.nih.gov/pubmed/?term=Boutet S%5BAuthor%5D&cauthor=true&cauthor_uid=15037732), [Earley K](https://www.ncbi.nlm.nih.gov/pubmed/?term=Earley K%5BAuthor%5D&cauthor=true&cauthor_uid=15037732), [Lawrence RJ](https://www.ncbi.nlm.nih.gov/pubmed/?term=Lawrence RJ%5BAuthor%5D&cauthor=true&cauthor_uid=15037732), [Pikaard CS](https://www.ncbi.nlm.nih.gov/pubmed/?term=Pikaard CS%5BAuthor%5D&cauthor=true&cauthor_uid=15037732), [Murfett J](https://www.ncbi.nlm.nih.gov/pubmed/?term=Murfett J%5BAuthor%5D&cauthor=true&cauthor_uid=15037732), [Furner I](https://www.ncbi.nlm.nih.gov/pubmed/?term=Furner I%5BAuthor%5D&cauthor=true&cauthor_uid=15037732), [Vaucheret H](https://www.ncbi.nlm.nih.gov/pubmed/?term=Vaucheret H%5BAuthor%5D&cauthor=true&cauthor_uid=15037732), [Mittelsten Scheid O](https://www.ncbi.nlm.nih.gov/pubmed/?term=Mittelsten Scheid O%5BAuthor%5D&cauthor=true&cauthor_uid=15037732): **Arabidopsis Histone Deacetylase HDA6 Is Required for Maintenance of Transcriptional Gene Silencing and Determines Nuclear Organization of rDNA Repeats.** [*Plant Cell*](https://www.ncbi.nlm.nih.gov/pubmed/?term=Arabidopsis+Histone+Deacetylase+HDA6+Is+Required+for+Maintenance+of+Transcriptional+Gene+Silencing+and+Determines+Nuclear+Organization+of+rDNA+Repeats.) 2004; **16(4)**:1021-34. doi:[10.1105/tpc.018754](https://dx.doi.org/10.1105/tpc.018754)
32. Zhang J, Guo X, Li X, Xiang F, Zhou B, Yu D, Tang D, Liu X. **The genetic and physiological analysis of late-flowering phenotype of T-DNA insertion mutants of AtCAL1 and AtCAL2 in Arabidopsis**. [*Mol Biol Rep*.](https://www.ncbi.nlm.nih.gov/pubmed/21695426) 2012; **39(2)**:1527-35. doi: 10.1007/s11033-011-0891-2
33. Han P, García-Ponce B, Fonseca-Salazar G, Alvarez-Buylla ER, and Yu H. **AGAMOUS-LIKE 17, a novel flowering promoter, acts in a FT-independent photoperiod pathway.** [*Plant J.*](https://www.ncbi.nlm.nih.gov/pubmed/18363787) 2008; **55(2)**: 253-65. doi: 10.1111/j.1365-313X.2008.03499.x.
34. [Hu YX](https://www.ncbi.nlm.nih.gov/pubmed/?term=Hu YX%5BAuthor%5D&cauthor=true&cauthor_uid=15040885), [Wang YX](https://www.ncbi.nlm.nih.gov/pubmed/?term=Wang YX%5BAuthor%5D&cauthor=true&cauthor_uid=15040885), [Liu XF](https://www.ncbi.nlm.nih.gov/pubmed/?term=Liu XF%5BAuthor%5D&cauthor=true&cauthor_uid=15040885), [Li JY](https://www.ncbi.nlm.nih.gov/pubmed/?term=Li JY%5BAuthor%5D&cauthor=true&cauthor_uid=15040885). **Arabidopsis RAV1 is down-regulated by brassinosteroid and may act as a negative regulator during plant development**. [*Cell Res.*](https://www.ncbi.nlm.nih.gov/pubmed/?term=Arabidopsis+RAV1+is+down-regulated+by+brassinosteroid+and+may+act+as+a+negative+regulator+during+plant+development.) 2004; **14(1)**: 8-15.
35. MacAlister CA, Ortiz-Ramírez C, Becker JD, Feijó JA, and Lippman ZB: **Hydroxyproline O-arabinosyltransferase mutants oppositely alter tip growth in Arabidopsis thaliana and Physcomitrella patens.** [*Plant J.*](https://www.ncbi.nlm.nih.gov/pubmed/?term=Hydroxyproline+O-arabinosyltransferase+mutants+oppositely+alter+tip+growth+in+Arabidopsis+thaliana+and+Physcomitrella+patens.) 2016; **85(2)**:193-208. doi: 10.1111/tpj.13079
36. E Z, Zhang Y, Li T, Wang L, Zhao H. **Characterization of the Ubiquitin-Conjugating Enzyme Gene Family in Rice and Evaluation of Expression Profiles under Abiotic Stresses and Hormone Treatments.** [*PLoS One*](https://www.ncbi.nlm.nih.gov/pubmed/?term=Characterization+of+the+Ubiquitin-Conjugating+Enzyme+Gene+Family+in+Rice+and+Evaluation+of+Expression+Profiles+under+Abiotic+Stresses+and+Hormone+Treatments.)2015; **10(4)**: e0122621. doi: 10.1371/journal.pone.0122621.
37. Ladwig F, Stahl M, Ludewig U, Hirner AA, Hammes UZ, Stadler R, Harter K, and Koch W. **Siliques Are Red1 from Arabidopsis Acts as a Bidirectional Amino Acid Transporter That Is Crucial for the Amino Acid Homeostasis of Siliques.** [*Plant Physiol.*](https://www.ncbi.nlm.nih.gov/pubmed/?term=Siliques+Are+Red1+from+Arabidopsis+Acts+as+a+Bidirectional+Amino+Acid+Transporter+That+Is+Crucial+for+the+Amino+Acid+Homeostasis+of+Siliques.) 2012; **158(4)**:1643-55. doi: 10.1104/pp.111.192583.
38. [Hwang SG](https://www.ncbi.nlm.nih.gov/pubmed/?term=Hwang SG%5BAuthor%5D&cauthor=true&cauthor_uid=22153241), [Lin NC](https://www.ncbi.nlm.nih.gov/pubmed/?term=Lin NC%5BAuthor%5D&cauthor=true&cauthor_uid=22153241), [Hsiao YY](https://www.ncbi.nlm.nih.gov/pubmed/?term=Hsiao YY%5BAuthor%5D&cauthor=true&cauthor_uid=22153241), [Kuo CH](https://www.ncbi.nlm.nih.gov/pubmed/?term=Kuo CH%5BAuthor%5D&cauthor=true&cauthor_uid=22153241), [Chang PF](https://www.ncbi.nlm.nih.gov/pubmed/?term=Chang PF%5BAuthor%5D&cauthor=true&cauthor_uid=22153241), [Deng WL](https://www.ncbi.nlm.nih.gov/pubmed/?term=Deng WL%5BAuthor%5D&cauthor=true&cauthor_uid=22153241), [Chiang MH](https://www.ncbi.nlm.nih.gov/pubmed/?term=Chiang MH%5BAuthor%5D&cauthor=true&cauthor_uid=22153241), [Shen HL](https://www.ncbi.nlm.nih.gov/pubmed/?term=Shen HL%5BAuthor%5D&cauthor=true&cauthor_uid=22153241), [Chen CY](https://www.ncbi.nlm.nih.gov/pubmed/?term=Chen CY%5BAuthor%5D&cauthor=true&cauthor_uid=22153241), [Cheng WH](https://www.ncbi.nlm.nih.gov/pubmed/?term=Cheng WH%5BAuthor%5D&cauthor=true&cauthor_uid=22153241): **The Arabidopsis short-chain dehydrogenase/reductase 3, an ABSCISIC ACID DEFICIENT 2 homolog, is involved in plant defense responses but not in ABA biosynthesis.** [*Plant Physiol Biochem.*](https://www.ncbi.nlm.nih.gov/pubmed/?term=.+The+Arabidopsis+short-chain+dehydrogenase%2Freductase+3%2C+an+ABSCISIC+ACID+DEFICIENT+2+homolog%2C+is+involved+in+plant+defense+responses+but+not+in+ABA+biosynthesis.) 2012; **51**:63-73. doi: 10.1016/j.plaphy.2011.10.013.
39. Ren J, Liu Z, Niu R, Feng H: **Mapping of Re, a gene conferring the red leaf trait in ornamental kale (Brassica oleracea L. var. acephala).** *Plant Breeding* 2015; [**134(4)**,](http://onlinelibrary.wiley.com/doi/10.1111/pbr.2015.134.issue-4/issuetoc) 494–500. doi: 10.1111/pbr.12286.
40. Moharekar S, Moharekar S, Tanaka R, Ogawa KI, Tanaka A, Hara T: **Great promoting effect of high irradiance from germination on flowering in Arabidopsis thaliana — a process of photo-acclimation.** [*Photosynthetica*](http://link.springer.com/journal/11099) 2007; **45(2)**, 259-265.
41. Zhang W, Gruszewski HA, Chevone BI, and Nessler CL: **An Arabidopsis Purple Acid Phosphatase with Phytase Activity Increases Foliar Ascorbate.** [*Plant Physiol.*](https://www.ncbi.nlm.nih.gov/pubmed/?term=An+Arabidopsis+Purple+Acid+Phosphatase+with+Phytase+Activity+Increases+Foliar+Ascorbate.) 2008; *146(2)*: 431-40.doi: [http:/​/​dx.​doi.​org/​10.​1104/​pp.​107.​109934](http://dx.doi.org/10.1104/pp.107.109934).
42. Turck F, Fornara F, and Couplan G: **Regulation and Identity of Florigen: FLOWERING LOCUS T Moves Center Stage.** [*Annu Rev Plant Biol.*](https://www.ncbi.nlm.nih.gov/pubmed/?term=Regulation+and+Identity+of+Florigen%3A+FLOWERING+LOCUS+T+Moves+Center+Stage.)2008; **59**: 573-94. doi: 10.1146/annurev.arplant.59.032607.092755.
43. [Wu K](https://www.ncbi.nlm.nih.gov/pubmed/?term=Wu K%5BAuthor%5D&cauthor=true&cauthor_uid=18212027), [Zhang L](https://www.ncbi.nlm.nih.gov/pubmed/?term=Zhang L%5BAuthor%5D&cauthor=true&cauthor_uid=18212027), [Zhou C](https://www.ncbi.nlm.nih.gov/pubmed/?term=Zhou C%5BAuthor%5D&cauthor=true&cauthor_uid=18212027), [Yu CW](https://www.ncbi.nlm.nih.gov/pubmed/?term=Yu CW%5BAuthor%5D&cauthor=true&cauthor_uid=18212027), [Chaikam V](https://www.ncbi.nlm.nih.gov/pubmed/?term=Chaikam V%5BAuthor%5D&cauthor=true&cauthor_uid=18212027): **HDA6 is required for jasmonate response, senescence and flowering in Arabidopsis.** [*J Exp Bot.*](https://www.ncbi.nlm.nih.gov/pubmed/?term=HDA6+is+required+for+jasmonate+response%2C+senescence+and+flowering+in+Arabidopsis) 2008; **59(2)**: 225-34. doi: 10.1093/jxb/erm300.
44. [Kim](http://www.sciencedirect.com/science/article/pii/S1097276511005284) TW, [Guan](http://www.sciencedirect.com/science/article/pii/S1097276511005284) S, [Burlingame](http://www.sciencedirect.com/science/article/pii/S1097276511005284) AL, [Wang](http://www.sciencedirect.com/science/article/pii/S1097276511005284) ZY: **The CDG1 Kinase Mediates Brassinosteroid Signal Transduction from BRI1 Receptor Kinase to BSU1 Phosphatase and GSK3-like Kinase BIN2.** [*Mol Cell.*](https://www.ncbi.nlm.nih.gov/pubmed/?term=The+CDG1+Kinase+Mediates+Brassinosteroid+Signal+Transduction+from+BRI1+Receptor+Kinase+to+BSU1+Phosphatase+and+GSK3-like+Kinase+BIN2.) 2011; **43(4)**: 561-71. doi: 10.1016/j.molcel.2011.05.037.
45. [Li](http://www.sciencedirect.com/science/article/pii/S1674205214609564) CL, [Wang](http://www.sciencedirect.com/science/article/pii/S1674205214609564) M, [Ma](http://www.sciencedirect.com/science/article/pii/S1674205214609564) XY, [Zhang](http://www.sciencedirect.com/science/article/pii/S1674205214609564) W: **NRGA1, a Putative Mitochondrial Pyruvate Carrier, Mediates ABA Regulation of Guard Cell Ion Channels and Drought Stress Responses in Arabidopsis.** [*Mol. Plant*](https://www.ncbi.nlm.nih.gov/pubmed/?term=NRGA1%2C+a+Putative+Mitochondrial+Pyruvate+Carrier%2C+Mediates+ABA+Regulation+of+Guard+Cell+Ion+Channels+and+Drought+Stress+Responses+in+Arabidopsis.) 2014; **7(10)**:1508-21. doi: 10.1093/mp/ssu061.
46. Yuan L, Liu X, Luo M, Yang S, and Wu K: [**Involvement of histone modifications in plant abiotic stress responses**](http://onlinelibrary.wiley.com/doi/10.1111/jipb.12060/full)**.** [*J. Integr Plant Biol.*](https://www.ncbi.nlm.nih.gov/pubmed/?term=Involvement+of+histone+modifications+in+plant+abiotic+stress+responses.) 2013; **55(10)**: 892-901. doi: 10.1111/jipb.12060.
47. Li X, Xu J, Yu G, Luo L: **A wound-induced small polypeptide gene family is upregulated in soybean nodules.** [*Chinese Science Bulletin*](http://link.springer.com/journal/11434), 2012; **58**[**(9)**,](http://link.springer.com/journal/11434/58/9/page/1) 1003-1009. doi: 10.1007/s11434-012-5495-2.
48. **Haerizadeh** F, **Wong** CE, **Bhalla** PL, **Gresshoff PM, Singh MB: Genomic expression profiling of mature soybean (Glycine max) pollen.**  [*BMC Plant Biol.*](https://www.ncbi.nlm.nih.gov/pubmed/?term=Genomic+expression+profiling+of+mature+soybean+(Glycine+max)++pollen.) 2009; **9**: 25. doi: 10.1186/1471-2229-9-25.
49. Piñeiro M, Gómez-Mena C, Schaffer R, Martínez-Zapater JM, and Coupland G: **EARLY BOLTING IN SHORT DAYS Is Related to Chromatin Remodeling Factors and Regulates Flowering in Arabidopsis by Repressing FT.** Plant Cell 2003; **15 (7)**, 1552-62. doi: [http:/​/​dx.​doi.​org/​10.​1105/​tpc.​012153](http://dx.doi.org/10.1105/tpc.012153).
50. [Tranquilli](http://jhered.oxfordjournals.org/search?author1=G+Tranquilli&sortspec=date&submit=Submit) G, [Dubcovsky](http://jhered.oxfordjournals.org/search?author1=J+Dubcovsky&sortspec=date&submit=Submit) J: **Epistatic interaction between vernalization genes Vrn-A^m^1 and Vrn-A^m^2 in diploid wheat.** [*J Hered.*](https://www.ncbi.nlm.nih.gov/pubmed/?term=Epistatic+interaction+between+vernalization+genes+Vrn-Am1+and+Vrn-Am2+in+diploid+wheat) 2000; **91(4)**: 304-6.
51. Wang H, Zhou Y, Gilmer S, Whitwill S, and Fowke CL: **Expression of the plant cyclin-dependent kinase inhibitor ICK1 affects cell division, plant growth and morphology.** [*Plant J.*](https://www.ncbi.nlm.nih.gov/pubmed/11123800) 2000; **24(5)**: 613-23. doi: 10.1046/j.1365-313x.2000.00899.x.
52. [Pesaresi](http://www.sciencedirect.com/science/article/pii/S000527281000678X) P, [Pribil](http://www.sciencedirect.com/science/article/pii/S000527281000678X) M, [Wunder](http://www.sciencedirect.com/science/article/pii/S000527281000678X) T, [Leiste](http://www.sciencedirect.com/science/article/pii/S000527281000678X)r D. **Dynamics of reversible protein phosphorylation in thylakoids of flowering plants: The roles of STN7, STN8 and TAP38.** [*Biochim Biophys Acta.*](https://www.ncbi.nlm.nih.gov/pubmed/?term=Dynamics+of+reversible+protein+phosphorylation+in+thylakoids+of+flowering+plants%3A+The+roles+of+STN7%2C+STN8+and+TAP38)2011; **1807(8)**: 887-96. doi: 10.1016/j.bbabio.2010.08.002..
53. Jacob Y, Feng S, LeBlanc CA, Bernatavichute YV, Stroud SH, Cokus S, Johnson LM, Pellegrini M, Jacobsen SE. & Michaels SD: **ATXR5 and ATXR6 are H3K27 mono methyltransferases required for chromatin structure and gene silencing.** [*Nat Struct Mol Biol.*](https://www.ncbi.nlm.nih.gov/pubmed/?term=ATXR5+and+ATXR6+are+H3K27+mono+methyltransferases+required+for+chromatin+structure+and+gene+silencing.) 2009; **16(7)**:763-8. doi: 10.1038/nsmb.1611..
54. [Yang ZT](https://www.ncbi.nlm.nih.gov/pubmed/?term=Yang ZT%5BAuthor%5D&cauthor=true&cauthor_uid=24961665), [Lu SJ](https://www.ncbi.nlm.nih.gov/pubmed/?term=Lu SJ%5BAuthor%5D&cauthor=true&cauthor_uid=24961665), [Wang MJ](https://www.ncbi.nlm.nih.gov/pubmed/?term=Wang MJ%5BAuthor%5D&cauthor=true&cauthor_uid=24961665), [Bi DL](https://www.ncbi.nlm.nih.gov/pubmed/?term=Bi DL%5BAuthor%5D&cauthor=true&cauthor_uid=24961665), [Sun L](https://www.ncbi.nlm.nih.gov/pubmed/?term=Sun L%5BAuthor%5D&cauthor=true&cauthor_uid=24961665), [Zhou SF](https://www.ncbi.nlm.nih.gov/pubmed/?term=Zhou SF%5BAuthor%5D&cauthor=true&cauthor_uid=24961665), [Song ZT](https://www.ncbi.nlm.nih.gov/pubmed/?term=Song ZT%5BAuthor%5D&cauthor=true&cauthor_uid=24961665), [Liu JX](https://www.ncbi.nlm.nih.gov/pubmed/?term=Liu JX%5BAuthor%5D&cauthor=true&cauthor_uid=24961665): **A plasma membrane-tethered transcription factor, NAC062/ANAC062/NTL6, mediates the unfolded protein response in Arabidopsis.** [*Plant J.*](https://www.ncbi.nlm.nih.gov/pubmed/?term=A+plasma+membrane-tethered+transcription+factor%2C+NAC062%2FANAC062%2FNTL6%2C+mediates+the+unfolded+protein+response+in+Arabidopsis.) 2014; **79(6)**: 1033-43. doi: 10.1111/tpj.12604.
55. Zhang J, Guo X, Li X, Xiang F, Zhou B, Yu D, Tang D, Liu X: **The genetic and physiological analysis of late-flowering phenotype of T-DNA insertion mutants of** AtCAL**1 and** AtCAL**2 in** Arabidopsi**s.** [Mol Biol Rep.](https://www.ncbi.nlm.nih.gov/pubmed/?term=The+genetic+and+physiological+analysis+of+late-flowering+phenotype+of+T-DNA+insertion+mutants+of+AtCAL1+and+AtCAL2+in+Arabidopsis.) 2012; **39(2)**:1527-35. doi: 10.1007/s11033-011-0891-2.
56. Applewhite BP, Kaur-Sawhney R, and Galston WA: **A role of spermidine in the bolting and flowering of Arabidopsis.** *Physiologia Plantarum.* 2000; **108**, 314–320. doi: 10.1034/j.1399-3054.2000.108003314.x.
57. Crocco CD, Holm M, Yanovsky MJ, Botto JF: **AtBBX21 and COP1 genetically interact in the regulation of shade avoidance.** *Plant J.* 2010; **64(4)**, 551–562. doi: 10.1111/j.1365-313X.2010.04360.x.
58. [Aalto MK](https://www.ncbi.nlm.nih.gov/pubmed/?term=Aalto MK%5BAuthor%5D&cauthor=true&cauthor_uid=22118612), [Helenius E](https://www.ncbi.nlm.nih.gov/pubmed/?term=Helenius E%5BAuthor%5D&cauthor=true&cauthor_uid=22118612), [Kariola T](https://www.ncbi.nlm.nih.gov/pubmed/?term=Kariola T%5BAuthor%5D&cauthor=true&cauthor_uid=22118612), [Pennanen V](https://www.ncbi.nlm.nih.gov/pubmed/?term=Pennanen V%5BAuthor%5D&cauthor=true&cauthor_uid=22118612), [Heino P](https://www.ncbi.nlm.nih.gov/pubmed/?term=Heino P%5BAuthor%5D&cauthor=true&cauthor_uid=22118612), [Hõrak H](https://www.ncbi.nlm.nih.gov/pubmed/?term=Hõrak H%5BAuthor%5D&cauthor=true&cauthor_uid=22118612), [Puzõrjova I](https://www.ncbi.nlm.nih.gov/pubmed/?term=Puzõrjova I%5BAuthor%5D&cauthor=true&cauthor_uid=22118612), [Kollist H](https://www.ncbi.nlm.nih.gov/pubmed/?term=Kollist H%5BAuthor%5D&cauthor=true&cauthor_uid=22118612), [Palva ET](https://www.ncbi.nlm.nih.gov/pubmed/?term=Palva ET%5BAuthor%5D&cauthor=true&cauthor_uid=22118612): **ERD15—An attenuator of plant ABA responses and stomatal aperture.** [*Plant Sci.*](https://www.ncbi.nlm.nih.gov/pubmed/?term=ERD15—An+attenuator+of+plant+ABA+responses+and+stomatal+aperture) 2012 ; **182**: 19-28. doi: 10.1016/j.plantsci.2011.08.009.
59. [Abe](http://pcp.oxfordjournals.org/search?author1=Makoto+Abe&sortspec=date&submit=Submit) M, [Fujiwara](http://pcp.oxfordjournals.org/search?author1=Masayuki+Fujiwara&sortspec=date&submit=Submit) M, [Kurotani](http://pcp.oxfordjournals.org/search?author1=Ken-ichi+Kurotani&sortspec=date&submit=Submit) K, [Yokoi](http://pcp.oxfordjournals.org/search?author1=Shuji+Yokoi&sortspec=date&submit=Submit)  S, and [Shimamoto](http://pcp.oxfordjournals.org/search?author1=Ko+Shimamoto&sortspec=date&submit=Submit) K. **Identification of Dynamin as an Interactor of Rice GIGANTEA by Tandem Affinity Purification (TAP)**. [*Plant Cell Physiol.*](https://www.ncbi.nlm.nih.gov/pubmed/?term=Identification+of+Dynamin+as+an+Interactor+of+Rice+GIGANTEA+by+Tandem+Affinity+Purification+(TAP)) 2008**; 49(3)**:420-32. doi: 10.1093/pcp/pcn019.
60. Wilson RN, Heckman JW, and Somerville CR. **Gibberellin Is Required for Flowering in Arabidopsis thaliana under Short Days.** *Plant Physiol.* 1992; **100(1)**, 403-408. doi: [http:/​/​dx.​doi.​org/​10.​1104/​pp.​100.​1.​403](http://dx.doi.org/10.1104/pp.100.1.403).
61. Kim HS, Abbasi N, Choi SB. **Bruno-like proteins modulate flowering time via 3′ UTR-dependent decay of SOC1 mRNA.** [*New Phytol.*](https://www.ncbi.nlm.nih.gov/pubmed/?term=Bruno-like+proteins+modulate+flowering+time+via+3′+UTR-dependent+decay+of+SOC1+mRNA) 2013; **198(3)**: 747-56. doi: 10.1111/nph.12181..
62. [Li](http://www.sciencedirect.com/science/article/pii/S1674205214609564) CL, [Wang](http://www.sciencedirect.com/science/article/pii/S1674205214609564) M, [Ma](http://www.sciencedirect.com/science/article/pii/S1674205214609564) XY, [Zhang](http://www.sciencedirect.com/science/article/pii/S1674205214609564) W: **NRGA1, a Putative Mitochondrial Pyruvate Carrier, Mediates ABA Regulation of Guard Cell Ion Channels and Drought Stress Responses in Arabidopsis.** [*Mol Plant.*](https://www.ncbi.nlm.nih.gov/pubmed/?term=NRGA1%2C+a+Putative+Mitochondrial+Pyruvate+Carrier%2C+Mediates+ABA+Regulation+of+Guard+Cell+Ion+Channels+and+Drought+Stress+Responses+in+Arabidopsis.) 2014; **7(10)**: 1508-21. doi: 10.1093/mp/ssu061..
63. [Pérez-Castro R](https://www.ncbi.nlm.nih.gov/pubmed/?term=Pérez-Castro R%5BAuthor%5D&cauthor=true&cauthor_uid=22247248), [Kasai K](https://www.ncbi.nlm.nih.gov/pubmed/?term=Kasai K%5BAuthor%5D&cauthor=true&cauthor_uid=22247248), [Gainza-Cortés F](https://www.ncbi.nlm.nih.gov/pubmed/?term=Gainza-Cortés F%5BAuthor%5D&cauthor=true&cauthor_uid=22247248), [Ruiz-Lara S](https://www.ncbi.nlm.nih.gov/pubmed/?term=Ruiz-Lara S%5BAuthor%5D&cauthor=true&cauthor_uid=22247248), [Casaretto JA](https://www.ncbi.nlm.nih.gov/pubmed/?term=Casaretto JA%5BAuthor%5D&cauthor=true&cauthor_uid=22247248), [Peña-Cortés H](https://www.ncbi.nlm.nih.gov/pubmed/?term=Peña-Cortés H%5BAuthor%5D&cauthor=true&cauthor_uid=22247248), [Tapia J](https://www.ncbi.nlm.nih.gov/pubmed/?term=Tapia J%5BAuthor%5D&cauthor=true&cauthor_uid=22247248), [Fujiwara T](https://www.ncbi.nlm.nih.gov/pubmed/?term=Fujiwara T%5BAuthor%5D&cauthor=true&cauthor_uid=22247248), [González E](https://www.ncbi.nlm.nih.gov/pubmed/?term=González E%5BAuthor%5D&cauthor=true&cauthor_uid=22247248): **VvBOR1, the Grapevine Ortholog of AtBOR1, Encodes an Efflux Boron Transporter That is Differentially Expressed Throughout Reproductive Development of Vitis vinifera L.** [*Plant Cell Physiol.*](https://www.ncbi.nlm.nih.gov/pubmed/?term=VvBOR1%2C+the+Grapevine+Ortholog+of+AtBOR1%2C+Encodes+an+Efflux+Boron+Transporter+That+is+Differentially+Expressed+Throughout+Reproductive+Development+of+Vitis+vinifera+L.) 2012 ; **53(2)**:485-94. doi: 10.1093/pcp/pcs001.
64. Bang W, Kim S, Ueda A, Vikram M, Yun D, Bressan RA, Hasegawa PM, Bahk J, and Koiwa H: **Arabidopsis Carboxyl-Terminal Domain Phosphatase-Like Isoforms Share Common Catalytic and Interaction Domains But Have Distinct in Planta Functions.** [*Plant Physiol.*](https://www.ncbi.nlm.nih.gov/pubmed/?term=Arabidopsis+Carboxyl-Terminal+Domain+Phosphatase-Like+Isoforms+Share+Common+Catalytic+and+Interaction+Domains+But+Have+Distinct+in+Planta+Functions.) 2006; **142(2)**: 586-94. doi: [http:/​/​dx.​doi.​org/​10.​1104/​pp.​106.​084939](http://dx.doi.org/10.1104/pp.106.084939).
65. [Schönrock N](https://www.ncbi.nlm.nih.gov/pubmed/?term=Schönrock N%5BAuthor%5D&cauthor=true&cauthor_uid=16778081), [Bouveret R](https://www.ncbi.nlm.nih.gov/pubmed/?term=Bouveret R%5BAuthor%5D&cauthor=true&cauthor_uid=16778081), [Leroy O](https://www.ncbi.nlm.nih.gov/pubmed/?term=Leroy O%5BAuthor%5D&cauthor=true&cauthor_uid=16778081), [Borghi L](https://www.ncbi.nlm.nih.gov/pubmed/?term=Borghi L%5BAuthor%5D&cauthor=true&cauthor_uid=16778081), [Köhler C](https://www.ncbi.nlm.nih.gov/pubmed/?term=Köhler C%5BAuthor%5D&cauthor=true&cauthor_uid=16778081), [Gruissem W](https://www.ncbi.nlm.nih.gov/pubmed/?term=Gruissem W%5BAuthor%5D&cauthor=true&cauthor_uid=16778081), [Hennig L](https://www.ncbi.nlm.nih.gov/pubmed/?term=Hennig L%5BAuthor%5D&cauthor=true&cauthor_uid=16778081): **Polycomb-group proteins repress the floral activator AGL19 in the FLC-independent vernalization pathway.** [*Genes Dev.*](https://www.ncbi.nlm.nih.gov/pubmed/?term=Polycomb-group+proteins+repress+the+floral+activator+AGL19+in+the+FLC-independent+vernalization+pathway) 2006; **20(12)**:1667-78. doi:10.1101/gad.377206.
66. [Engstrom EM](https://www.ncbi.nlm.nih.gov/pubmed/?term=Engstrom EM%5BAuthor%5D&cauthor=true&cauthor_uid=21173022), [Andersen CM](https://www.ncbi.nlm.nih.gov/pubmed/?term=Andersen CM%5BAuthor%5D&cauthor=true&cauthor_uid=21173022), [Gumulak-Smith J](https://www.ncbi.nlm.nih.gov/pubmed/?term=Gumulak-Smith J%5BAuthor%5D&cauthor=true&cauthor_uid=21173022), [Hu J](https://www.ncbi.nlm.nih.gov/pubmed/?term=Hu J%5BAuthor%5D&cauthor=true&cauthor_uid=21173022), [Orlova E](https://www.ncbi.nlm.nih.gov/pubmed/?term=Orlova E%5BAuthor%5D&cauthor=true&cauthor_uid=21173022), [Sozzani R](https://www.ncbi.nlm.nih.gov/pubmed/?term=Sozzani R%5BAuthor%5D&cauthor=true&cauthor_uid=21173022), [Bowman JL](https://www.ncbi.nlm.nih.gov/pubmed/?term=Bowman JL%5BAuthor%5D&cauthor=true&cauthor_uid=21173022): **Arabidopsis homologs of the petunia hairy meristem gene are required for maintenance of shoot and root indeterminacy.** [*Plant Physiol.*](https://www.ncbi.nlm.nih.gov/pubmed/?term=Arabidopsis+Homologs+of+the+PetuniaHAIRY+MERISTEM+Gene+Are+Required+for+Maintenance+of+Shoot+and+Root+Indeterminacy) 2011; **155(2)**: 735-50. doi: 10.1104/pp.110.
67. [Murmu J](https://www.ncbi.nlm.nih.gov/pubmed/?term=Murmu J%5BAuthor%5D&cauthor=true&cauthor_uid=20805327), [Bush MJ](https://www.ncbi.nlm.nih.gov/pubmed/?term=Bush MJ%5BAuthor%5D&cauthor=true&cauthor_uid=20805327), [DeLong C](https://www.ncbi.nlm.nih.gov/pubmed/?term=DeLong C%5BAuthor%5D&cauthor=true&cauthor_uid=20805327), [Li S](https://www.ncbi.nlm.nih.gov/pubmed/?term=Li S%5BAuthor%5D&cauthor=true&cauthor_uid=20805327), [Xu M](https://www.ncbi.nlm.nih.gov/pubmed/?term=Xu M%5BAuthor%5D&cauthor=true&cauthor_uid=20805327), [Khan M](https://www.ncbi.nlm.nih.gov/pubmed/?term=Khan M%5BAuthor%5D&cauthor=true&cauthor_uid=20805327), [Malcolmson C](https://www.ncbi.nlm.nih.gov/pubmed/?term=Malcolmson C%5BAuthor%5D&cauthor=true&cauthor_uid=20805327), [Fobert PR](https://www.ncbi.nlm.nih.gov/pubmed/?term=Fobert PR%5BAuthor%5D&cauthor=true&cauthor_uid=20805327), [Zachgo S](https://www.ncbi.nlm.nih.gov/pubmed/?term=Zachgo S%5BAuthor%5D&cauthor=true&cauthor_uid=20805327), [Hepworth SR](https://www.ncbi.nlm.nih.gov/pubmed/?term=Hepworth SR%5BAuthor%5D&cauthor=true&cauthor_uid=20805327). **Arabidopsis Basic Leucine-Zipper Transcription Factors TGA9 and TGA10 Interact with Floral Glutaredoxins ROXY1 and ROXY2 and Are Redundantly Required for Anther Development.** [*Plant Physiol.*](https://www.ncbi.nlm.nih.gov/pubmed/?term=Arabidopsis+Basic+Leucine-Zipper+Transcription+Factors+TGA9+and+TGA10+Interact+with+Floral+Glutaredoxins+ROXY1+and+ROXY2+and+Are+Redundantly+Required+for+Anther+Development.) 2010; **154(3)**:1492-504. doi: 10.1104/pp.110.159111.
68. [Jorgensen](http://www.sciencedirect.com/science/article/pii/S1055790314000487) SA, [Preston](http://www.sciencedirect.com/science/article/pii/S1055790314000487) JC: **Differential SPL gene expression patterns reveal candidate genes underlying flowering time and architectural differences in Mimulus and Arabidopsis.** [*Mol Phylogenet Evol.*](https://www.ncbi.nlm.nih.gov/pubmed/?term=Differential+SPL+gene+expression+patterns+reveal+candidate+genes+underlying+flowering+time+and+architectural+differences+in+Mimulus+and+Arabidopsis.+Molecular+Phylogenetics+and+Evolution) 2014; **73**: 129-39. doi: 10.1016/j.ympev.2014.01.029.
69. Shaw LM, Turner AS, Herry L, Griffiths S, Laurie DA: **Mutant Alleles ofPhotoperiod-1 in Wheat (Triticum aestivum L.) That Confer a Late Flowering Phenotype in Long Days.** [*PLoS One.*](https://www.ncbi.nlm.nih.gov/pubmed/?term=Mutant+Alleles+ofPhotoperiod-1+in+Wheat+(Triticum+aestivum+L.)+That+Confer+a+Late+Flowering+Phenotype+in+Long+Days.) 2013; **8(11)**: e79459. doi: 10.1371/journal.pone.0079459.
70. [Shearman](http://www.sciencedirect.com/science/article/pii/S0888754313000414) JR, [Antasuriyarat](http://www.sciencedirect.com/science/article/pii/S0888754313000414) C,  [Sangsrakru](http://www.sciencedirect.com/science/article/pii/S0888754313000414) D, [Yoocha](http://www.sciencedirect.com/science/article/pii/S0888754313000414) T, [Vannavichit](http://www.sciencedirect.com/science/article/pii/S0888754313000414) A,  [Tragoonrung](http://www.sciencedirect.com/science/article/pii/S0888754313000414) S, [Tangphatsornruang](http://www.sciencedirect.com/science/article/pii/S0888754313000414) S: **Transcriptome analysis of normal and mantled developing oil palm flower and fruit.** [*Genomics*](https://www.ncbi.nlm.nih.gov/pubmed/?term=Transcriptome+analysis+of+normal+and+mantled+developing+oil+palm+flower+and+fruit.)2013; **101(5)**: 306-12. doi: 10.1016/j.ygeno.2013.02.012.
71. [Sjögren LL](https://www.ncbi.nlm.nih.gov/pubmed/?term=Sjögren LL%5BAuthor%5D&cauthor=true&cauthor_uid=16980539), [Stanne TM](https://www.ncbi.nlm.nih.gov/pubmed/?term=Stanne TM%5BAuthor%5D&cauthor=true&cauthor_uid=16980539), [Zheng B](https://www.ncbi.nlm.nih.gov/pubmed/?term=Zheng B%5BAuthor%5D&cauthor=true&cauthor_uid=16980539), [Sutinen S](https://www.ncbi.nlm.nih.gov/pubmed/?term=Sutinen S%5BAuthor%5D&cauthor=true&cauthor_uid=16980539), [Clarke AK](https://www.ncbi.nlm.nih.gov/pubmed/?term=Clarke AK%5BAuthor%5D&cauthor=true&cauthor_uid=16980539): **Structural and Functional Insights into the Chloroplast ATP-Dependent Clp Protease in Arabidopsis*.*** [*Plant Cell.*](https://www.ncbi.nlm.nih.gov/pubmed/?term=Structural+and+Functional+Insights+into+the+Chloroplast+ATP-Dependent+Clp+Protease+in+Arabidopsis.) 2006; **18(10)**: 2635-49. doi: [http:/​/​dx.​doi.​org/​10.​1105/​tpc.​106.​044594](http://dx.doi.org/10.1105/tpc.106.044594).
72. Colombo M, Masiero S, Vanzulli S, Lardelli P, Kater MM, Colombo L: **AGL23, a type I MADS-box gene that controls female gametophyte and embryo development in Arabidopsis**. [*Plant J*.](https://www.ncbi.nlm.nih.gov/pubmed/?term=AGL23%2C+a+type+I+MADS-box+gene+that+controls+female+gametophyte+and+embryo+development+in+Arabidopsis) 2008; **54(6)**: 1037-48. doi: 10.1111/j.1365-313X.2008.03485.x.
73. [Kim HU](https://www.ncbi.nlm.nih.gov/pubmed/?term=Kim HU%5BAuthor%5D&cauthor=true&cauthor_uid=15772283), [Li Y](https://www.ncbi.nlm.nih.gov/pubmed/?term=Li Y%5BAuthor%5D&cauthor=true&cauthor_uid=15772283), [Huang AH](https://www.ncbi.nlm.nih.gov/pubmed/?term=Huang AH%5BAuthor%5D&cauthor=true&cauthor_uid=15772283): **Ubiquitous and endoplasmic reticulum-located lysophosphatidyl acyltransferase, LPAT2, is essential for female but not male gametophyte development in Arabidopsis.** [*Plant Cell*.](https://www.ncbi.nlm.nih.gov/pubmed/?term=Ubiquitous+and+endoplasmic+reticulum-located+lysophosphatidyl+acyltransferase%2C+LPAT2%2C+is+essential+for+female+but+not+male+gametophyte+development+in+Arabidopsis) 2005; **17(4)**:1073-89.
74. [Nagano](http://pcp.oxfordjournals.org/search?author1=Atsushi+J.+Nagano&sortspec=date&submit=Submit) AJ, [Fukao](http://pcp.oxfordjournals.org/search?author1=Yoichiro+Fukao&sortspec=date&submit=Submit) Y, [Fujiwara](http://pcp.oxfordjournals.org/search?author1=Masayuki+Fujiwara&sortspec=date&submit=Submit) M, [Nishimura](http://pcp.oxfordjournals.org/search?author1=Mikio+Nishimura&sortspec=date&submit=Submit) M, and [Hara-Nishimura](http://pcp.oxfordjournals.org/search?author1=Ikuko+Hara-Nishimura&sortspec=date&submit=Submit) I: **Antagonistic Jacalin-Related Lectins Regulate the Size of ER Body-Type β-Glucosidase Complexes in Arabidopsis thaliana.** [*Plant Cell Physiol.*](https://www.ncbi.nlm.nih.gov/pubmed/?term=Antagonistic+Jacalin-Related+Lectins+Regulate+the+Size+of+ER+Body-Type+β-Glucosidase+Complexes+in+Arabidopsis+thaliana.) 2008; **49(6)**: 969-80. doi: 10.1093/pcp/pcn075..
75. [Adam](http://www.sciencedirect.com/science/article/pii/S0005273613001594) Z: **Emerging roles for diverse intramembrane proteases in plant biology.** [*Biochim Biophys Acta*.](https://www.ncbi.nlm.nih.gov/pubmed/?term=Emerging+roles+for+diverse+intramembrane+proteases+in+plant+biology) 2013; **1828(12)**: 2933-6. doi: 10.1016/j.bbamem.2013.05.013.
76. Trigueros M, Navarrete-Gómez M, Sato S, Christensen SK, Pelaz S, Weigel D, Yanofsky MF, and Ferrándiz C: **The NGATHA Genes Direct Style Development in the Arabidopsis Gynoecium.** [*Plant Cel*](https://www.ncbi.nlm.nih.gov/pubmed/19435937)*l*2009; **21(5)**: 1394-409. doi: 10.1105/tpc.109.065508.
77. **Chen P, Jäger G, and  Zheng B: Transfer RNA modifications and genes for modifying enzymes in** **Arabidopsis thaliana**. [*BMC Plant Biol.*](https://www.ncbi.nlm.nih.gov/pubmed/?term=Transfer+RNA+modifications+and+genes+for+modifying+enzymes+in+Arabidopsis+thaliana) 2010;**10**:201. doi: 10.1186/1471-2229-10-201.
78. Han YF, Huang HW, Li L, Cai T, Chen S, He XJ: **The Cytosolic Iron-Sulfur Cluster Assembly Protein MMS19 Regulates Transcriptional Gene Silencing, DNA Repair, and Flowering Time in** Arabidopsis**.** *PLoS ONE.* 2015; **10(6)**, e0129137. doi:10.1371/journal.pone.0129137.
79. Li S, Fu Q, Huang W, and Yu D: **Functional analysis of an Arabidopsis transcription factor WRKY25 in heat stress. Biotic and Abiotic Stress.** [*Plant Cell Reports*](http://link.springer.com/journal/299) 2009; **28(4)**, 683-693. doi: 10.1007/s00299-008-0666-y.
80. Tolin S, Arrigoni G, Trentin AR, Veljovic-Jovanovic S, Pivato M, Zechman B, Masi A: **Biochemical and quantitative proteomics investigations in Arabidopsis ggt1 mutant leaves reveal a role for the gamma-glutamyl cycle in plant's adaptation to environment.** *Proteomics* 2013; **13(12-13)**, 2031–45. doi: 10.1002/pmic.201200479.
81. [Liu LJ](https://www.ncbi.nlm.nih.gov/pubmed/?term=Liu LJ%5BAuthor%5D&cauthor=true&cauthor_uid=18296627), [Zhang YC](https://www.ncbi.nlm.nih.gov/pubmed/?term=Zhang YC%5BAuthor%5D&cauthor=true&cauthor_uid=18296627), [Li QH](https://www.ncbi.nlm.nih.gov/pubmed/?term=Li QH%5BAuthor%5D&cauthor=true&cauthor_uid=18296627), [Sang Y](https://www.ncbi.nlm.nih.gov/pubmed/?term=Sang Y%5BAuthor%5D&cauthor=true&cauthor_uid=18296627), [Mao J](https://www.ncbi.nlm.nih.gov/pubmed/?term=Mao J%5BAuthor%5D&cauthor=true&cauthor_uid=18296627), [Lian HL](https://www.ncbi.nlm.nih.gov/pubmed/?term=Lian HL%5BAuthor%5D&cauthor=true&cauthor_uid=18296627), [Wang L](https://www.ncbi.nlm.nih.gov/pubmed/?term=Wang L%5BAuthor%5D&cauthor=true&cauthor_uid=18296627), [Yang HQ](https://www.ncbi.nlm.nih.gov/pubmed/?term=Yang HQ%5BAuthor%5D&cauthor=true&cauthor_uid=18296627): **COP1-Mediated Ubiquitination of CONSTANS Is Implicated in Cryptochrome Regulation of Flowering in** Arabidopsis. [*Plant Cell*](https://www.ncbi.nlm.nih.gov/pubmed/?term=Mediated+Ubiquitination+of+CONSTANS+Is+Implicated+in+Cryptochrome+Regulation+of+Flowering+in+Arabidopsis.) 2008; **20(2)**: 292-306. doi: 10.1105/tpc.107.057281.
82. [Sugano](http://www.pnas.org/search?author1=Shoji+Sugano&sortspec=date&submit=Submit) S, [Andronis](http://www.pnas.org/search?author1=Christos+Andronis&sortspec=date&submit=Submit) C, [Ong](http://www.pnas.org/search?author1=May+S.+Ong&sortspec=date&submit=Submit) MS, [Green](http://www.pnas.org/search?author1=Rachel+M.+Green&sortspec=date&submit=Submit) RM, and [Tobin](http://www.pnas.org/search?author1=Elaine+M.+Tobin&sortspec=date&submit=Submit) EM: **The protein kinase CK2 is involved in regulation of circadian rhythms in** Arabidopsis. [*Proc Natl Acad Sci U S A.*](https://www.ncbi.nlm.nih.gov/pubmed/10535927)1999; **96(22)**: 12362-6. doi: 10.1073/pnas.96.22.12362.
